# A Multivariable Decision Rule for Weight-Rocking-Driven Onset Detection Method Bias on Bilateral Force Plates Across 32,952 Countermovement Jump Trials

**DOI:** 10.3390/s26165043

**Published:** 2026-08-08

**Authors:** Bahman Adlou, Michael D. Goodlett, Christopher Wilburn, Wendi Weimar

**Affiliations:** 1 School of Kinesiology, Auburn University, Auburn, AL 36849, USA; 2 Department of Kinesiology, School of Health and Human Sciences, University of North Carolina at Greensboro, Greensboro, NC 27412, USA; 3 Athletics Department, Auburn University, Auburn, AL 36849, USA

**Keywords:** movement-onset detection, postural sway, body weight estimation, jump height reliability, decision curve analysis

## Abstract

Bilateral vertical ground reaction force (vGRF) plates are the dominant sensor for field-deployed neuromuscular monitoring in athletes, with the countermovement jump (CMJ) being the dominant test. Detecting movement onset on the vGRF trace is a single decision that propagates through impulse–momentum integration to bias jump height. Existing onset method comparisons used fewer than 100 athletes, and bilateral weight rocking, a form of pre-jump postural sway during quiet standing, has not been characterized at scale as a method-specific source of disagreement. We analyzed 32,952 NCAA Division I CMJ trials from 579 athletes across 15 teams recorded on bilateral vGRF plates. Five hypotheses were pre-specified, and a per-trial decision rule was developed under leave-one-athlete-out cross-validation. Pooled, 45.89% of trials showed false-early firing on at least one of five non-first-derivative onset methods, relative to a rate-of-change reference, at a 30 ms threshold. On the rocking-amplified subset, a bidirectional body weight band method overestimated jump height by 0.943 cm versus the reference. The decision rule reached an area under the receiver operating characteristic curve of 0.71 with near-perfect calibration. It is a per-trial screening flag, not a deterministic classifier, and deployment should anchor on balanced-accuracy or high-precision operating points, holding the onset method constant across an athlete’s monitoring timeline.

## 1. Introduction

Bilateral vertical ground reaction force (vGRF) plates are the dominant sensor architecture for field-deployed neuromuscular monitoring in elite athletes, with commercial implementations from VALD ForceDecks, Hawkin Dynamics, Sparta Science, and others deployed across professional, collegiate, and military training environments [1]. The countermovement jump (CMJ) is the dominant signal acquisition protocol on this sensor class, with applications spanning training-load periodization, fatigue monitoring, return-to-play decision-making, and longitudinal performance characterization [2,3,4,5]. A forward-dynamics impulse–momentum estimate of jump height integrates the system-mass-normalized vGRF signal from movement onset to takeoff, and any discrepancy at the onset detection step propagates through the integration to bias jump height, takeoff velocity, and every phase duration metric anchored to the onset [5,6]. The validity of these field-derived neuromuscular metrics therefore depends on the validity of the onset detection method applied to the sensor output and on whether that method is robust to the population-specific signal characteristics encountered in routine field deployment. The validity of these field-derived metrics has, however, been examined mainly in homogeneous samples and rarely in relation to the population-specific signal characteristics encountered across multiple sports.

The number of existing comparison studies of CMJ onset detection methods has remained small. Meylan et al. (2011) compared three percentage-of-body-weight thresholds in 10 soccer players and reported that threshold choice altered braking and concentric kinetics by clinically relevant margins [6]. Owen et al. [7] established the now-canonical five-standard-deviation (SD) rule with a 30 ms back-step in 15 male professional rugby players. Pinto and Callaghan (2023) showed that the First-derivative method with a 10 Hz low-pass filter produced the tightest limits of agreement against manually selected onsets [8]. Barefoot et al. (2022) compared four threshold methods in 21 college-aged participants and implemented each threshold in both an above-or-below and a below-only form, because threshold crossing can occur above the system weight before unweighting begins; the two forms produced significantly different net impulse, jump height, and peak power [9]. Park et al. (2025) compared four start detection methods in 22 collegiate male basketball players performing the CMJ with arm swing and recommended a band anchored to the maximum and minimum of the weighing-phase force (BW = body weight; max BW + 2.5% or min BW − 2.5%, hereafter Range-BW), which produced the lowest erroneous start-identification rate of 3.03% [10]. The cumulative participant pool across the existing body of work is limited, and no study has explicitly modeled bilateral weight rocking during pre-jump quiet standing as a method-specific source of onset detection discrepancy.

Quiet bipedal standing is not a static state. Anteroposterior excursions during quiet standing are dominated by an ankle strategy and medio-lateral excursions by a hip load–unload strategy when the feet are placed side by side [11], and the sway itself is actively generated rather than passively damped, with the soleus and gastrocnemius maintaining balance through ballistic-like predictive adjustments rather than spring-like stiffness [12,13,14]. The literature characterizes sway in the horizontal plane; the vertical-axis force fluctuation that necessarily accompanies center-of-mass excursion has not been characterized during the pre-jump weighing phase. Postural threat reduces sway amplitude and increases sway frequency [15], and asymmetrical body weight distribution produces greater center-of-pressure displacement under each foot [16]. Inter-limb force asymmetry persists into elite athlete populations, with asymmetry magnitude associated with reduced physical and sports performance across athlete populations [17], two-legged CMJ asymmetries being unrelated to one-legged CMJ asymmetries [18], and lower-extremity asymmetry patterns extending across strength and vertical ground reaction force metrics in elite soccer [19]. Quiet-standing postural balance is also associated with CMJ performance in athlete populations, with the relationship being weak overall and moderated by training history [20].

Despite this body of literature, vertical-axis rocking amplitudes, frequencies, and incidence rates during the CMJ weighing phase have not been cataloged in elite athlete populations, and per-sport variation in pre-jump postural noise has not been documented at scale. Adlou et al. [21] reported, on visual inspection of trial-level outputs from a 32,952-trial multi-sport CMJ dataset, that bilateral weight rocking during pre-movement quiet standing produces low-amplitude force oscillation that crosses permissive onset thresholds before the true countermovement begins. This mechanism was identified as being most pronounced on the Range-BW threshold of Park et al. [10] and progressively less pronounced on SD-, percentage-, and fixed-absolute-anchored thresholds, with the First-derivative method of Pinto and Callaghan [8] least affected, yet a systematic characterization was deferred. Adjacent to this gap, decision curve analysis [22,23,24] has become standard for clinical prediction model evaluation but has not been applied to force-plate-derived CMJ outcomes or to onset detection classification, and the degeneracy of net-benefit-maximization at low threshold probabilities in high-prevalence binary outcomes has not been formally addressed in the sports biomechanics literature.

The present study provides systematic characterization of bilateral weight rocking as a method-specific source of onset detection discrepancy in CMJ force–time analysis using a 32,952-trial NCAA Division I dataset across 15 teams and 12 sports. Five hypotheses were pre-specified:

**Hypothesis** **1** **(H1)** **(incidence).** *Rocking is present in at least 5% of dataset-wide trials (tested in Section 3.2).*

**Hypothesis** **2** **(H2)** **(method-ranking).** *Range-BW is the most false-early-prone threshold method on the vertical-fluctuation axis (tested in Section 3.2 and Supplementary Table S2).*

**Hypothesis** **3** **(H3)** **(downstream).** *When Range-BW fires earlier than the reference on a rocking-amplified trial, the resulting jump height difference is positive (tested in Section 3.4).*

**Hypothesis** **4** **(H4)** **(sport modifier).** *Sports with higher Poor quiet standing proportions exhibit higher rocking incidence (tested in Section 3.3).*

**Hypothesis** **5** **(H5)** **(decision rule).** *A multivariable rule discriminates trials whose absolute Range-BW versus First-derivative jump height difference reaches 0.50 cm (tested in Section 3.6).*

This study contributes (i) the first large-scale characterization of bilateral weight rocking as an onset detection discrepancy source on a dataset of order 10^4^ trials, (ii) a multivariable decision rule for per-trial onset method selection with operating-point coordinates and reference method noise sensitivity, and (iii) a constructive observation about operating-point selection in base-rate-balanced sports biomechanics classification.

## 2. Materials and Methods

### 2.1. Dataset and Processing Pipeline

This study analyzed 32,952 bilateral countermovement jump (CMJ) trials collected from 579 NCAA Division I athletes across 15 teams and 12 sports between 2021 and 2025 at a single athletics department, contributed across 11,410 testing sessions (Table 1).

Trials were recorded using VALD ForceDecks FDMax bilateral force plates (VALD Performance, Brisbane, Australia) at native sampling rates of 250 Hz (*n* = 768, 2.33%), 500 Hz (*n* = 15,279, 46.37%), or 1000 Hz (*n* = 16,905, 51.30%). Records were retrieved via the VALD Performance API as a deidentified export. The Institutional Review Board approved access to deidentified data (Protocol #STUDY00000694). The arms-akimbo CMJ protocol specified three trials per session; pre-jump quiet standing duration was determined by each athlete’s testing cadence managed by the VALD software rather than by an externally enforced timer. Sex assignment at the individual level was available for 544 athletes; 35 athletes from Diving (*n* = 16) and Track (*n* = 19) were sex-unresolved at the individual level per the deidentified data retrieval protocol.

VALD ForceDecks FDMax acquires only the vertical-axis ground reaction force (vGRF) on each of two bilateral plates and reports their sum as $F_{z,\mathrm{total}}$. No anterior–posterior or medio-lateral shear-force channels exist in the exported data. Medio-lateral weight transfer between plates is observable through the bilateral force share; vertical-axis fluctuations are observable through the summed signal; and pure anterior–posterior postural sway with negligible vertical coupling is largely invisible to this hardware. The three rocking-detection signals defined in Section 2.2 are vGRF-derived surrogates of rocking, and their scope follows from this sensor-class constraint.

Trial segmentation, BW estimation, and movement-onset detection followed the procedure of Adlou et al. [21], in which 32,952 trials were processed through 30 pipeline variants combining five BW estimators and six onset detection methods (First-derivative with 10 Hz low-pass filter, [8]; 5 × SD with 30 ms back-step, [7]; 4 × SD without back-step, [9]; Range-BW at 2.5% envelope, [10]; Percentage-BW at 2.5% envelope; Absolute 20 N envelope). A self-contained summary of the inherited segmentation, BW estimation, onset detection, quiet-standing tier, and plausibility-filtering procedures, sufficient to reproduce the present analyses without the companion paper, is given in Supplementary Section S1. The sliding min-SD BW row served as the canonical estimator throughout. A two-stage plausibility filter [21] excluded a trial method row when the multi-method engine failed to extract required landmarks (R4) or when derived quantities fell outside the literature-anchored bounds (R5) on jump height (0.05 to 0.80 m), takeoff velocity (0.99 to 4.0 m s^−1^), countermovement depth (−0.60 to −0.05 m), unweighting duration (50 to 1500 ms), braking duration (30 to 600 ms), and propulsion duration (50 to 600 ms).

**Figure 1:**
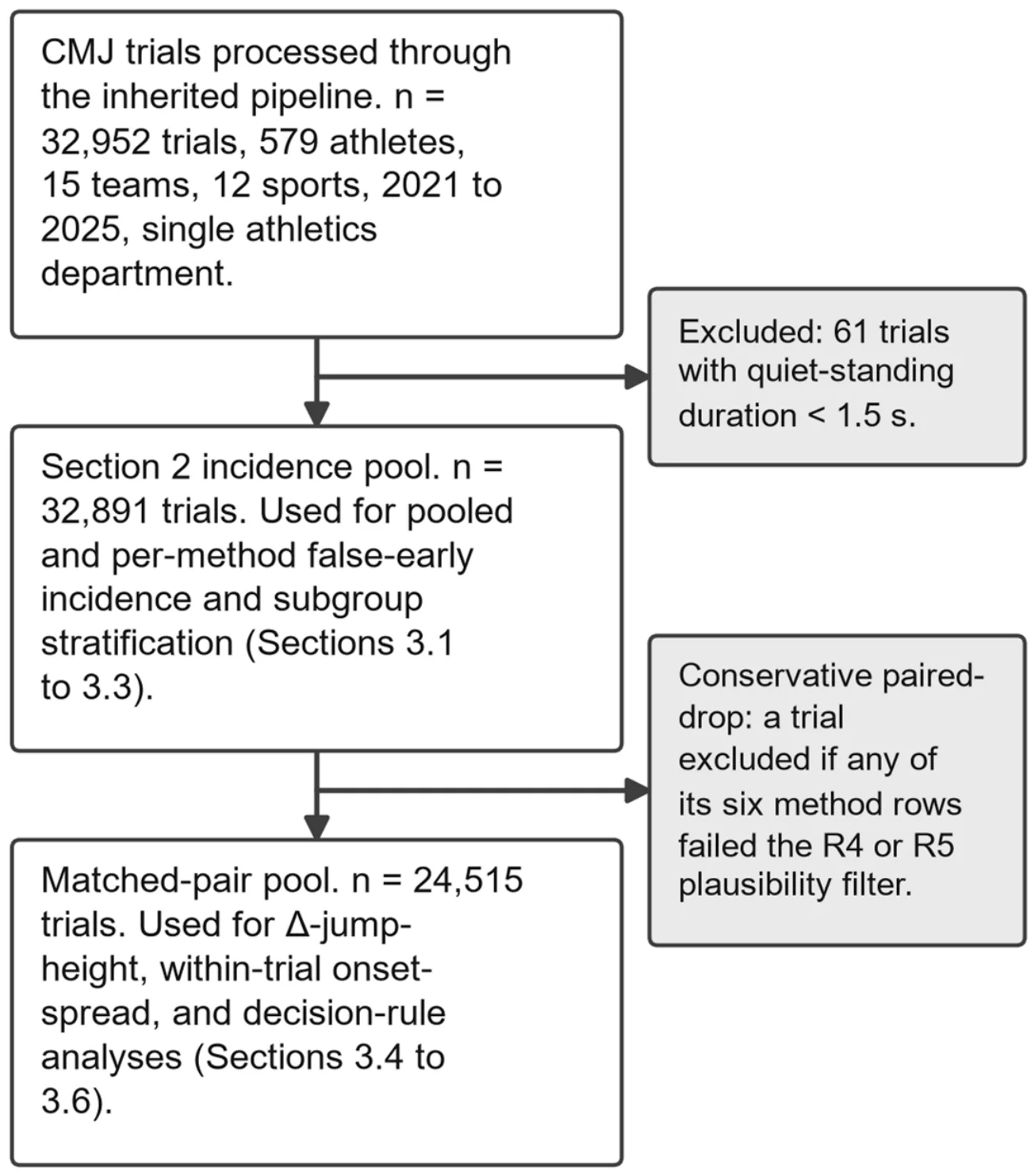
Trial flow from the inherited processing pipeline to the two analysis incidence pools. Of 32,952 processed countermovement jump trials (579 athletes, 15 teams, 12 sports, 2021 to 2025, single athletics department), 61 trials with quiet-standing duration below 1.5 s were excluded, leaving the *n* = 32,891 pool used for incidence and per-method analyses. A conservative paired-drop rule, which excluded a trial if any of its six method rows failed the R4 or R5 plausibility filter, yielded the *n* = 24,515 matched-pair pool used for the jump height, within-trial onset-spread, and decision-rule analyses. Within the matched-pair pool, the Candidate-A-high and Candidate-B-high tertiles contained 7262 and 7141 trials, respectively.

Two further filters were applied for the present analyses. First, a minimum quiet-standing (QS) duration of 1.5 s was required, set by the slowest of the three rocking-detection signals (the 1 s sliding-SD signal of Section 2.2) plus a 0.5 s edge-effect buffer. This filter excluded 61 trials, yielding the *n* = 32,891 incidence pool (Section 3.1, Section 3.2 and Section 3.3). Second, a conservative paired-drop rule excluded a trial from matched-pair Δ–jump height and within-trial onset-spread analyses if any of its six method rows failed R4 or R5, yielding the *n* = 24,515 matched-pair pool used in Section 3.4, Section 3.5 and Section 3.6. Figure 1 summarizes this trial flow.

### 2.2. Rocking-Detection Signals

Three QS signals were computed per trial. Each trial’s QS window was the half-open interval [$t_{min},\;t_{\mathrm{onset}}$), where $t_{\mathrm{onset}}$ is the segmentation onset. Candidate A is the peak-to-peak (max minus min) of the bilateral force share $f_{\mathrm{left}}=F_{z,\mathrm{left}}/F_{z,\mathrm{total}}$ over the QS window after a 0.5 s rolling-median smoothing. Candidate B is the maximum of the sliding 1 s standard deviation of $F_{z,\mathrm{total}}$ across the QS window, in newtons. Candidate C is the count of zero-crossings per second of the linearly detrended $F_{z,\mathrm{total}}$ across the QS window, in Hz. All three signals are computed at the trial’s native sampling rate without resampling.

The three candidates serve mechanistically distinct analytical roles. Candidate A is the directly observable form of rocking on bilateral 1D-plate hardware and is used as the headline incidence axis. Candidate B is the operational predictor for the Range-BW false-early row because Range-BW thresholds against summed vGRF, which pure medio-lateral rocking conserves; vertical-axis fluctuation captured by Candidate B is the direct mechanism for Range-BW false-early firing. Candidate C is reported for periodicity discrimination as a sensitivity check. For binned cross-tabulations, each signal was discretized into low, medium, and high tertiles using cutpoints derived from the in-sample distribution on the *n* = 32,891 pool. Candidate-B tertile cutpoints were 6.18 and 18.14 N (range 1.48 to 1377.20 N).

### 2.3. Operational Definitions

For each trial, each non-first-derivative onset method M was compared against the trial’s First-derivative onset on the same row under the canonical sliding min-SD BW estimator. Method *M*’s onset was classified as false-early at threshold Δ if and only if

(1) $$t_{\mathrm{onset}}^{\left(M\right)}\;<\;t_{\mathrm{onset}}^{\left(\mathrm{FD}\right)}\;-\;\Delta .$$

The primary effect-size threshold was Δ = 30 ms, anchored on the 30 ms back-step correction in the 5 × SD method [7] and on the 30 ms criterion-validity tolerance for First-derivative agreement with manually identified onsets [8]. A secondary Δ = 50 ms sensitivity threshold was reported. First-derivative served as the operational onset reference for three reasons. First, it is the most closely valid method against externally identified onsets. Pinto and Callaghan (2023) reported that a First-derivative method with a 10 Hz low-pass filter produced the tightest limits of agreement against manually selected onsets among the methods they compared [8]. Second, it is mechanistically the least susceptible to pre-movement postural fluctuation. Since it responds to the rate of change in force rather than to a threshold crossing, the low-frequency quiet-standing sway documented above produces only a small rate of change relative to the ballistic unweighting, whereas a threshold method crosses a static force band whose effective margin is widened by sway amplitude. Third, it is the only method in the comparison set that does not threshold against a quantity directly perturbed by the rocking surrogates of Section 2.2. We therefore treat First-derivative as the best available operational reference, not as an infallible ground truth, and we are careful to distinguish method disagreement from onset detection error throughout. We use the term false-early to denote detection within the pre-movement quiet-standing window, earlier than this reference. This determination does not depend on the reference being exactly correct, because the quiet-standing window precedes any genuine unweighting departure; a threshold crossing within it reflects a response to postural fluctuation rather than to movement onset. Barefoot et al. (2022) reached an analogous conclusion in an independent sample, attributing threshold crossings that occurred above system weight during weighing to weight-shift artifact rather than to unweighting onset [9].

In addition, each trial was assigned a QS quality tier (Good, Acceptable, Poor) using the classifier of Adlou et al. [21], with cutpoints anchored at the within-window standard deviation of summed vertical force over the 1 s sliding minimum-SD window: Good (≤5 N), Acceptable (5 < SD ≤ 10 N), Poor (>10 N). The analytic frame inherited the per-trial QS-Quality-Tier directly from the source pipeline output, with a runtime cross-check assertion at every team-load step. The incidence pool’s distribution was Good 65.12%, Acceptable 16.72%, Poor 18.15%, reproducing the distribution reported by Adlou et al. [21] within 0.05 percentage points.

### 2.4. Downstream-Consequence Analyses

The proposed five hypotheses were pre-specified. H1 (incidence): rocking is present at ≥5% of trials dataset-wide; refutation floor 0.5% (the pre-specified incidence-floor check). H2 (method-ranking): Range-BW is the most false-early-prone threshold method, tested on Candidate B per the axis-bifurcation rationale of Section 2.2. H3 (downstream): when Range-BW fires falsely early, the resulting jump height bias is positive, anchored on the matched-pair Range-BW-versus-first-derivative jump height bias of +0.20 cm reported by Adlou et al. [21]. H4 (sport modifier): sports with higher Poor-QS proportions exhibit higher rocking incidence, tested as Spearman rank correlation across the 15 sports. H5 (decision rule): a multivariable rule combining the rocking signals with QS-window duration and QS-tier discriminates trials with $\left|\Delta \mathrm{JH}\right|\geq 0.50$ cm.

The downstream-consequence pipeline operates on the matched-pair pool (*n* = 24,515) under the canonical sliding min-SD BW row. The headline reporting unit is a Bland–Altman analysis of paired Range-BW versus First-derivative jump height on the rocking-affected Candidate-B-high subset, with mean bias, 95% bootstrap confidence interval, 95% limits of agreement, and proportional-bias slope. A four-row BW-method sensitivity (Supplementary Table S5) confirms robustness to BW-estimator choice. Three derived metrics are reported: jump height (impulse–momentum), takeoff velocity, and modified Reactive-Strength Index (RSImod). Phase duration Δ-values are reported in Supplementary Table S3. A per-trial sign-consistency flag records whether the five non-first-derivative methods’ Δ–jump height values share a common sign within trial.

Two pre-specified checks operationalize H3 falsification. The magnitude check fires if the median absolute Range-BW jump height difference in the Candidate-A-high subset falls below 0.50 cm. This 0.50 cm floor is a conservative fraction, approximately one-third, of the smallest worthwhile change for countermovement jump height reported in athlete populations (approximately 1.3 to 1.5 cm; [25,26]), chosen so that a positive label captures method disagreements that are at least a meaningful fraction of the smallest worthwhile change rather than trivial numerical noise [27]. The direction check fires if the median signed Range-BW jump height difference in the Candidate-B-high subset is negative, contradicting the H3 directional prediction.

### 2.5. Decision-Rule Analyses

The decision rule targets a binary per-trial outcome (Label B). A trial is positive when the absolute Range-BW versus First-derivative paired Δ–jump height equals or exceeds 0.50 cm and is negative otherwise. Pooled Label B prevalence in the matched-pair pool is 0.4594. The decision context for the rule is per-trial onset method selection by the analyst processing CMJ force–time data. For a given trial, the rule’s predicted probability that the absolute Range-BW versus First-derivative jump height difference reaches the 0.50 cm threshold is used, at a chosen operating point, to decide whether to override the default onset method (apply First-derivative in place of Range-BW) or to flag the trial for manual onset inspection. The decision-maker is the sport scientist or biomechanist; the action is an onset method override or a manual review flag; the input is the per-trial predicted probability. There are four continuous (Candidates A, B, and C; QS-window duration in seconds) and one categorical (QS-tier, three levels with Good as reference) predictor. A predictor-diagnostic gate confirmed acceptable multicollinearity (max |Spearman ρ| = 0.668; max VIF = 3.58). Because trials are clustered within athletes, the pooled incidence and the headline jump height difference are additionally reported with an athlete-clustered bootstrap that resamples athletes with replacement and retains all of each resampled athlete’s trials (1000 resamples); the decision rule is evaluated under cross-validation grouped by athlete identifier, which already respects this clustering.

All five hypotheses and their falsification checks are specified before analysis, which constrains analytic flexibility, and exact *p*-values are reported throughout so that readers may apply a multiplicity correction of their choice; a Bonferroni correction across the five primary hypotheses (α = 0.01) does not alter any conclusion reported here.

Three model specifications were evaluated under an eligibility-then-AIC tournament. M1 is linear logistic with continuous predictors entered linearly and QS-tier dummies. M2 discretizes Candidate B into in-sample tertile dummies with the remaining predictors being linear. M3 is a 3-knot restricted cubic spline on Candidate B with knots placed at the 10th, 50th, and 90th percentiles per [28]. A specification was eligible only if all 20 leave-one-athlete-out (LOAO) folds converged with finite mean training-fold AIC and valid operating-point coordinates on the pooled $n_{\mathrm{test}}$ = 24,515. Among eligible specifications, the lowest mean training-fold AIC was selected as the headline rule.

The primary cross-validation scheme was K = 20 grouped folds over the deidentified athlete identifier; K = 20 grouped over the testing session identifier and K = 12 leave-one-sport-out (LOSO) were reported as sensitivity checks. Three pre-specified operating points on the M1 ROC curve are reported. Youden-J is the balanced-misclassification anchor [29]. PPV ≥ 0.70 is the deployment anchor. NB-max at $p_{t}\;$= 0.15 is the decision curve anchor [22,24], with bracket thresholds at $p_{t}$ = 0.10 and $p_{t}$ = 0.20. Calibration was reported via the slope and intercept of pooled LOAO predicted probabilities against the decile-bin observed positive rate.

Two sensitivity sub-analyses operationalized the First-derivative-as-noisy-reference framing of Section 2.3. Variant 1 perturbed the per-trial First-derivative onset by Gaussian noise at σ ∈ {5, 10} ms, recomputed Label B, and refit M1. Variant 2 replaced the First-derivative onset with a leave-one-method-out (LOMO) consensus reference computed as the per-trial median across the other five methods. Variant 3 (LOSO portability of M1) is reported in Supplementary Section S4.

Four pre-specified checks with literature-anchored cutpoints are reported; the full anchoring rationale is tabulated in Supplementary Table S1. The discrimination check fires if pooled LOAO AUC falls below 0.65 with the bootstrap upper CI below 0.70 [30]. The practical-utility check fires if the PPV at NB-max at $p_{t}\;$= 0.15 falls below 0.50 with a PPV-to-prevalence ratio below 2.0 [23,24]. The reference-robustness checks (Gaussian-noise variant and consensus-reference variant) fire if the AUC drop under Variant 1 or Variant 2 exceeds the Hopkins [27] trivial-versus-small effect-size boundary of 0.05 on the AUC scale.

Net benefit at threshold probability $p_{t}$ is NB = (TP/N) − (FP/N) × $p_{t}$/(1 − $p_{t}$), where TP and FP are the true- and false-positive counts at the operating point and $p_{t}$/(1 − $p_{t}$) is the false-positive penalty weight; the treat-all reference is p − (1 − p) × $p_{t}$/(1 − $p_{t}$) at outcome prevalence *p*. The derivation of the low-$p_{t}$ convergence at the prevailing prevalence, with the model and reference net benefit tabulated across $p_{t}$, is given in Supplementary Section S3.

Analyses were performed in Python 3.12.10 (Python Software Foundation, Wilmington, DE, USA) using pandas 3.0.2, NumPy 2.4.4, SciPy 1.17.1, Matplotlib 3.10.8, statsmodels 0.14.6, and Patsy 1.0.2. Analysis code is available from the corresponding author on reasonable request.

## 3. Results

### 3.1. Distributions of the Rocking-Detection Signals

**Figure 2:**
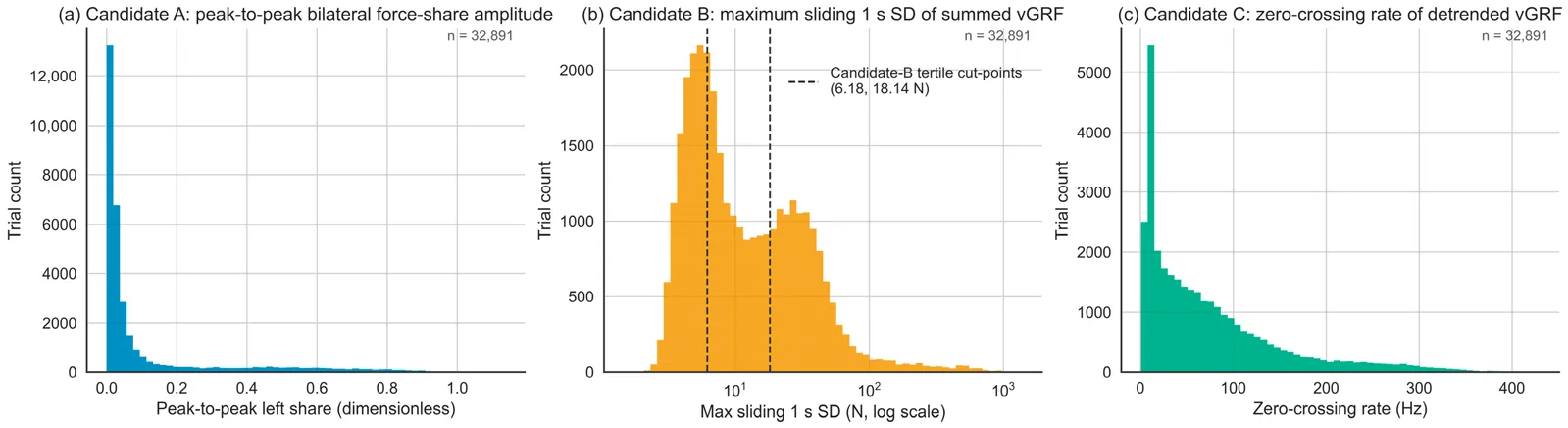
Distributions of the three rocking-detection signals across the *n* = 32,891 incidence pool. Panel (**a**): Candidate A (peak-to-peak bilateral-force-share oscillation amplitude after 0.5 s rolling-median smoothing, dimensionless); panel (**b**): Candidate B (maximum sliding 1 s standard deviation of summed vertical ground reaction force across the QS window, in newtons); panel (**c**): Candidate C (zero-crossing rate of detrended summed vertical ground reaction force across the QS window, in Hz). Vertical dashed lines on panel (**b**) mark the in-sample Candidate-B tertile cutpoints (6.18 N and 18.14 N). Panel (**b**)’s horizontal axis is shown on a logarithmic scale for legibility of the upper tail.

The three rocking-detection signals were computed on all 32,891 trials in the incidence pool (Figure 2). Candidate A was heavy-tailed, with most mass concentrated in the 0 to 0.05 dimensionless interval (median ≈ 0.02) and a long upper tail extending to approximately 0.95. Candidate B was heavily right-skewed, with a sharp mode in the 0 to 25 N interval and an upper tail extending to 1377.20 N. Tertile cutpoints on the incidence pool were 6.18 and 18.14 N. Candidate C was moderately right-skewed, peaking in the 30 to 60 Hz interval and extending to approximately 400 Hz. The three distributions are mechanistically distinct rather than being three estimates of one rocking magnitude, supporting the analytical-role assignment of Section 2.2.

### 3.2. Pooled and Per-Method False-Early-Onset Incidence

Across the 32,891 trials in the incidence pool, the pooled any-method false-early-onset rate at Δ = 30 ms was 45.89% (95% bootstrap confidence interval [45.4%, 46.4%]); in roughly four of every nine trials, at least one of the five non-first-derivative methods fired more than 30 ms earlier than the trial’s First-derivative reference. At the secondary Δ = 50 ms threshold, the rate was 45.32%, indicating that the false-early signal was robustly populated rather than noise-driven near the operational threshold. The pre-specified incidence-floor check was not fired; observed incidence exceeded the floor by approximately two orders of magnitude. Under an athlete-clustered bootstrap that accounted for the clustering of trials within athletes, the pooled any-method false-early rate was 45.89% (athlete-clustered 95% confidence interval: 44.31 to 47.58), still approximately two orders of magnitude above the 0.5% floor. The clustered interval is roughly three times wider than the trial-level interval, reflecting that the effective sample size is smaller than the nominal trial count (mean 56.8 trials per athlete), but the conclusion is unchanged.

Per-method incidence rates at Δ = 30 ms are reported in Table 2. Among the five threshold-anchored methods, 4 × SD without back-step produced the highest false-early rate (38.07%), followed by 5 × SD (29.24%), Absolute 20 N (28.58%), and Percentage-BW (28.10%). The Range-BW method produced the *lowest* non-first-derivative rate (7.67%), inverting the original H2 expectation that Range-BW would be the most rocking-fragile method.

**Figure 3:**
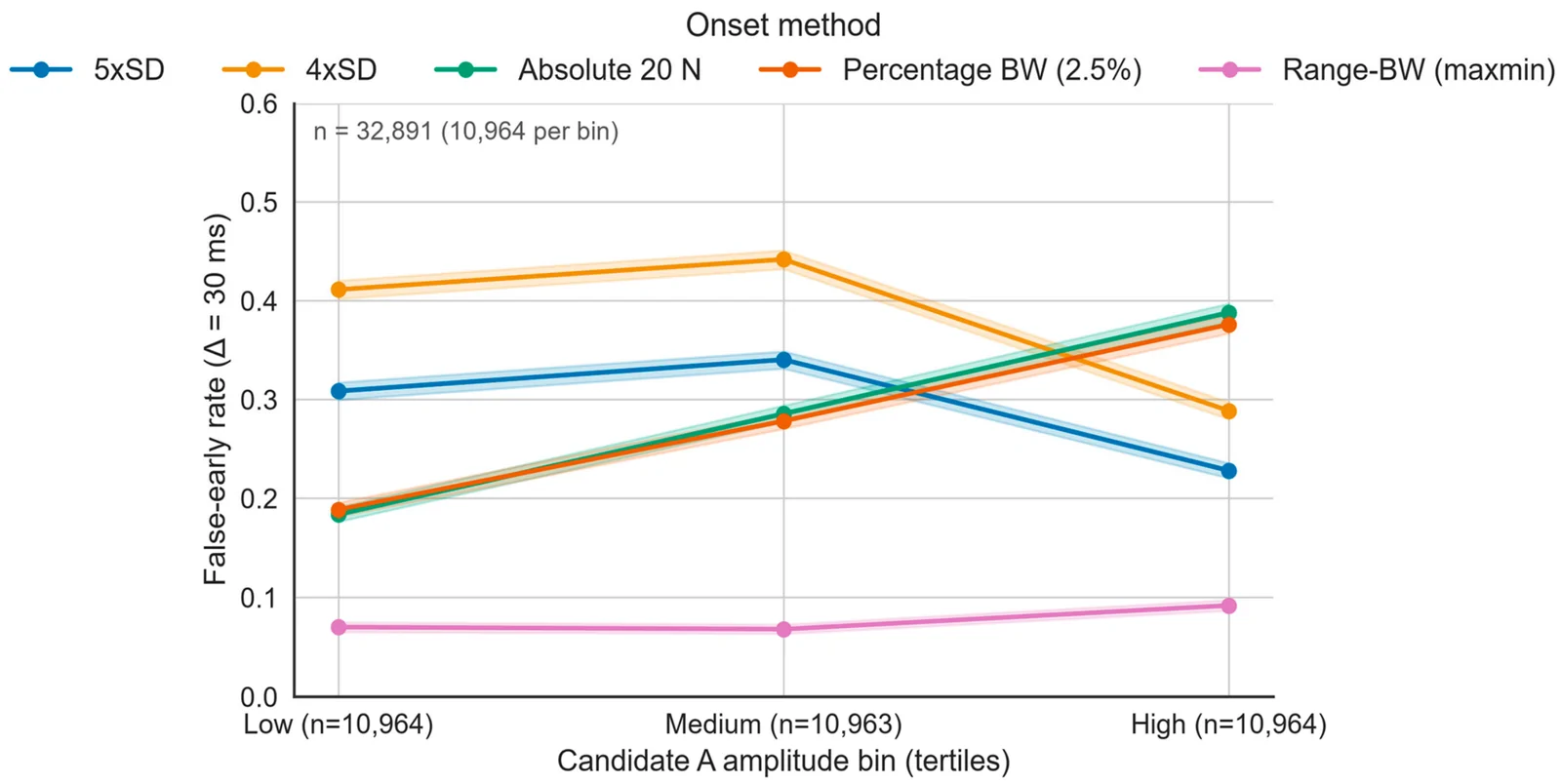
Per-method false-early-onset rate at Δ = 30 ms as a function of Candidate-A bilateral-force-share oscillation amplitude bin (low, medium, high tertiles), reported across the *n* = 32,891 incidence pool. Five non-first-derivative methods are shown: 4 × SD without back-step, 5 × SD with 30 ms back-step, Absolute 20 N envelope, Percentage-BW at 2.5%, and Range-BW at 2.5%. Error bars indicate 95% bootstrap confidence intervals. Three method classes are visible: monotonically increasing (Percentage-BW and Absolute 20 N), high-bin drop (4 × SD and 5 × SD), and approximately flat (Range-BW). The flat Range-BW pattern reflects the hardware-geometry argument of Section 2.2.

Per-bin patterns across the Candidate-A tertiles (Figure 3) revealed three method classes. Percentage-BW and Absolute 20 N rates increased monotonically with Candidate-A amplitude from 18.85% (low) to 27.83% (medium) to 37.60% (high) for Percentage-BW, and from 18.37% to 28.56% to 38.80% for Absolute 20 N. The 4 × SD and 5 × SD methods produced similar rates in the low and medium bins but substantially lower rates in the high bin (4 × SD: 41.13%/44.19%/28.87%; 5 × SD: 30.86%/34.04%/22.81%). The Range-BW rate was approximately flat across all three Candidate-A bins (7.01%/6.80%/9.19%).

The flat pattern for Range-BW across Candidate A is consistent with the hardware-geometry argument of Section 2.2. Pure medio-lateral weight transfer conserves summed vGRF, so Candidate-A amplitude and Range-BW false-early firing are decoupled by hardware. The mechanism-relevant predictor of Range-BW false-early firing is Candidate B, and the corresponding Candidate-B-binned analysis is reported in Supplementary Table S2. On the Candidate-B axis, Range-BW false-early firing rises modestly but significantly from the low to high tertile (6.65% to 8.13%, non-overlapping confidence intervals) yet remains the lowest of the five non-first-derivative methods on every bin, whereas Percentage-BW and Absolute 20 N rise steeply to approximately 40%. The analysis therefore confirms the axis distinction that motivated the comparison, because Range-BW responds to the vertical-fluctuation surrogate rather than the medio-lateral surrogate, but it does not support H2 in its original ordering form. Range-BW is the least false-early-prone threshold method, and its mechanistically distinctive behavior is its bias direction (Section 3.4) rather than its firing frequency.

### 3.3. Subgroup Stratification

**Figure 4:**
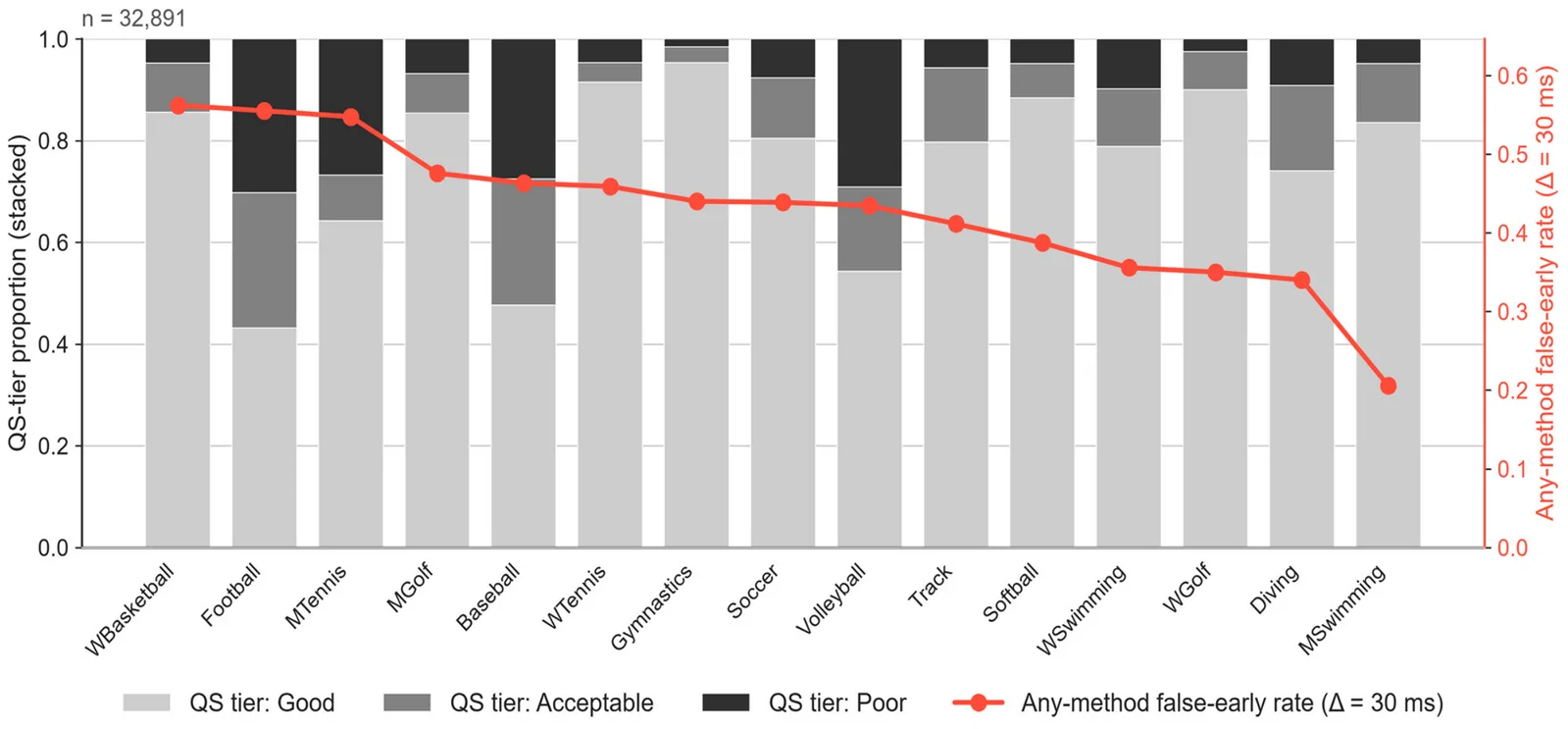
Per-sport any-method false-early-onset rate at Δ = 30 ms ordered by descending rate, with stacked Good/Acceptable/Poor quiet-standing tier composition overlaid on each sport bar. The two diagnostic anomalies for the H4 test, Women’s Basketball and Volleyball, are labeled in red on the horizontal axis; the red line and right-hand axis show the any-method false-early-onset rate. The 15-sport Spearman rank correlation between per-sport Poor-QS proportion and per-sport any-method false-early-onset rate is ρ = 0.218 (Section 3.3).

The rocking signature was present in every sport, both sexes, and every native sampling rate. Per-sport any-method false-early-onset rates at Δ = 30 ms ranged from 20.55% (Men’s Swimming) to 56.21% (Women’s Basketball), with every sport exceeding 20% (Table 3 and Figure 4); the Range-BW column alone ranged from 0.52% (Women’s Swimming) to 10.27% (Football).

The pre-specified Spearman rank correlation between per-sport Poor-QS proportion and per-sport any-method false-early-onset rate was ρ = 0.218 (95% bootstrap CI: −0.34 to +0.72; *p* = 0.435; *n* = 15 sports), and the corresponding Range-BW column correlation was numerically identical at this sample size; the Pearson sensitivity check supported the same conclusion (Supplementary Table S7). H4 was not statistically supported; the positive sign was directionally consistent but the correlation was weak and non-significant. Two anomalies, Women’s Basketball (4.73% Poor-QS, 56.21% rocking incidence) and Volleyball (29.15% Poor-QS, 43.45% rocking incidence), illustrate that Poor-QS proportion is one of several modifiers rather than a dominant predictor of per-sport rocking incidence.

Per-sex any-method incidence at Δ = 30 ms was 48.83% in male athletes (*n* = 16,139), 43.64% in female athletes (*n* = 15,102), and 37.88% in sex-unresolved Diving and Track athletes (*n* = 1650). The 5.19-percentage-point male–female difference is a between-sport contrast labeled by sex rather than a within-sport sex effect, because the 12 sports partition into wholly disjoint men-only and women-only programs.

Per-sampling-rate any-method incidence at Δ = 30 ms was 46.22% at 250 Hz, 50.54% at 500 Hz, and 41.66% at 1000 Hz. The cross-rate range of approximately nine percentage points is non-monotonic, indicating that rocking is a physical signature of pre-jump quiet standing rather than an acquisition-rate artifact.

### 3.4. Downstream Jump Height Bias

The matched-pair pool (*n* = 24,515) was reduced from the *n* = 32,891 incidence pool by the conservative paired-drop rule. Within this pool, the Candidate-A-high tertile contained *n* = 7262 trials and the Candidate-B-high tertile contained *n* = 7141 trials.

**Figure 5:**
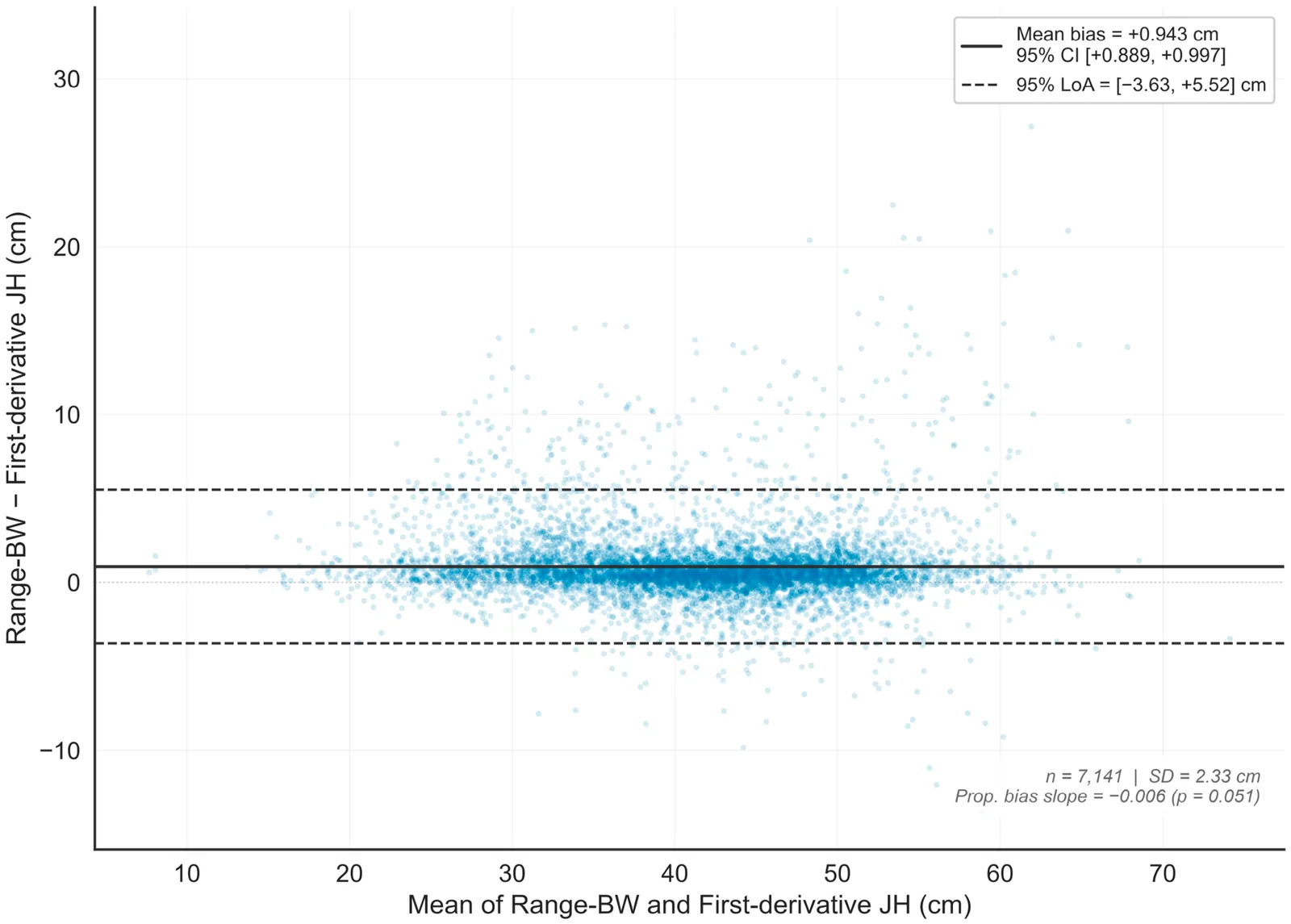
Bland–Altman plot of Range-BW (maxmin_bw_2p5) versus First-derivative jump height in the Candidate-B-high subset of the matched-pair pool (*n* = 7141). Mean bias = +0.943 cm (95% bootstrap confidence interval: +0.889 to +0.997); 95% limits of agreement = −3.63 to +5.52 cm; standard deviation of paired differences = 2.33 cm. The horizontal dashed line at the mean bias and the upper and lower dotted lines at ±1.96 SD demarcate the limits of agreement. The proportional-bias regression slope is −0.006 (*p* = 0.051), indicating no significant proportional dependence of bias on the paired mean jump height.

Within the Candidate-B-high subset (Figure 5), Range-BW jump height was higher than First-derivative jump height on the same trial by a mean bias of +0.943 cm (95% bootstrap confidence interval: +0.889 to +0.997). The 95% limits of agreement were −3.63 to +5.52 cm (standard deviation of paired differences = 2.33 cm). The proportional-bias regression slope was −0.006 (*p* = 0.051), which was not significant at the conventional α = 0.05 cutoff. Compared with the dataset-wide matched-pair Range-BW-versus-first-derivative jump height bias of +0.20 cm reported by Adlou et al. [21] on 142,336 plausibly bounded matched pairs, the Candidate-B-high subset showed a mean bias approximately 4.7-fold larger in the predicted direction. Under the athlete-clustered bootstrap, the mean bias was +0.943 cm (athlete-clustered 95% confidence interval: +0.795 to +1.110), still excluding zero and still above the 0.50 cm threshold, with the clustered interval roughly three times wider than the trial-level interval.

Both pre-specified checks were evaluated. The direction check tests whether the Candidate-B-amplified subset shows the predicted positive bias direction. The median signed paired difference on the Candidate-B-high subset was +0.5897 cm (95% CI: +0.5663 to +0.6148); the CI was fully above zero, and the direction check was not fired. The magnitude check tests whether the rocking-affected bias exceeds the Hopkins-anchored 0.50 cm floor. Median absolute paired difference on the Candidate-A-high subset was 0.6139 cm (95% CI: 0.5983 to 0.6344); the CI was fully above the threshold, and the magnitude check was not fired.

The H3 directional prediction holds in aggregate for Range-BW but does not extend in its naive form to the four other non-first-derivative threshold methods. Within the Candidate-A-high subset (Table 4), only Range-BW produced an aggregate positive jump height bias (median of +0.342 cm, mean of +0.589 cm). The four other threshold methods all produced aggregate negative jump height biases. The Wilcoxon signed-rank test against zero rejected the null at *p* < 0.001 for all five non-first-derivative methods. The per-method sign asymmetry (Range-BW positive and the other four threshold methods negative) is mechanistically distinct from the simple H3 prediction and motivates the within-trial sign-consistency analysis reported next.

The Range-BW takeoff velocity bias on the Candidate-A-high subset had a median of +0.012 m s^−1^ and mean of +0.021 m s^−1^, consistent with the impulse–momentum identity $\mathrm{JH}=v_{\mathrm{to}}^{2}/\left(2g\right)$ that constrains takeoff velocity and jump height biases to share a sign. Range-BW RSImod bias had a median of +0.065 and mean of +0.084.

### 3.5. Within-Trial Onset Spread and Sign Consistency

The within-trial onset spread, defined as max minus min onset time across the six methods within each trial, had a median of 134 ms (95th percentile: 894 ms) across the matched-pair pool. The absolute magnitude is itself a practical concern, exceeding the operational Δ = 30 ms threshold of Section 3.2 by more than four-fold. Spearman rank correlations between the within-trial onset spread and rocking-detection signal magnitude on both rocking axes were weak (|ρ| < 0.20) with mixed directions; full numerics are reported in Supplementary Table S6. A single-team Volleyball pilot of this analysis returned ρ = +0.325 on within-team data, an estimate that did not replicate at the all-teams scale, illustrating the importance of all-teams-scale aggregation for headline statistical claims in multi-team biomechanics datasets. Within-trial onset disagreement is large in absolute terms but is not monotonically driven by rocking magnitude on either axis.

The proportion of trials with within-trial sign-inconsistent paired Δ–jump height (Table 5) decreased monotonically with rocking-detection signal magnitude on both axes. On the Candidate-A axis, the proportion fell from 66.98% in the low bin to 61.01% in the medium bin and 52.84% in the high bin; on the Candidate-B axis, it fell from 66.07% to 57.86% to 57.06%. The dose–response change is steeper on Candidate A (14.14-percentage-point drop from low to high) than on Candidate B (9.01-percentage-point drop from low to high).

### 3.6. Decision-Rule Operating Characteristics

Among the three pre-specified candidate model forms, only the linear logistic specification (M1) and the Candidate-B-tertile-discretized specification (M2) satisfied the eligibility gate. The restricted-cubic-spline specification (M3) did not converge on 19 of 20 leave-one-athlete-out folds owing to numerical instability in the spline basis and was disqualified. Among the two eligible specifications, M1 produced a mean training-fold AIC of 28,797.96 versus 29,403.85 for M2 (ΔAIC = 605.89 in favor of M1), and M1 was selected as the headline specification.

Pooled leave-one-athlete-out discrimination on the matched-pair pool was AUC = 0.7112 (95% bootstrap CI: 0.7045 to 0.7174), with AUPRC = 0.6812 (CI: 0.6736 to 0.6889) against a no-skill baseline of 0.4594. Both the observed AUC and its upper confidence limit exceed the no-discrimination-versus-acceptable cutpoint of Hosmer et al. [30]; the discrimination check is not fired. An AUC of 0.71 represents moderate discrimination. The rule is therefore a per-trial decision aid and screening flag rather than a deterministic classifier or a replacement for visual onset inspection; at the balanced operating point it provides a 36% precision uplift over the base rate, and at the high-precision operating point it identifies approximately 41% of consequential trials at approximately 70% precision.

**Figure 6:**
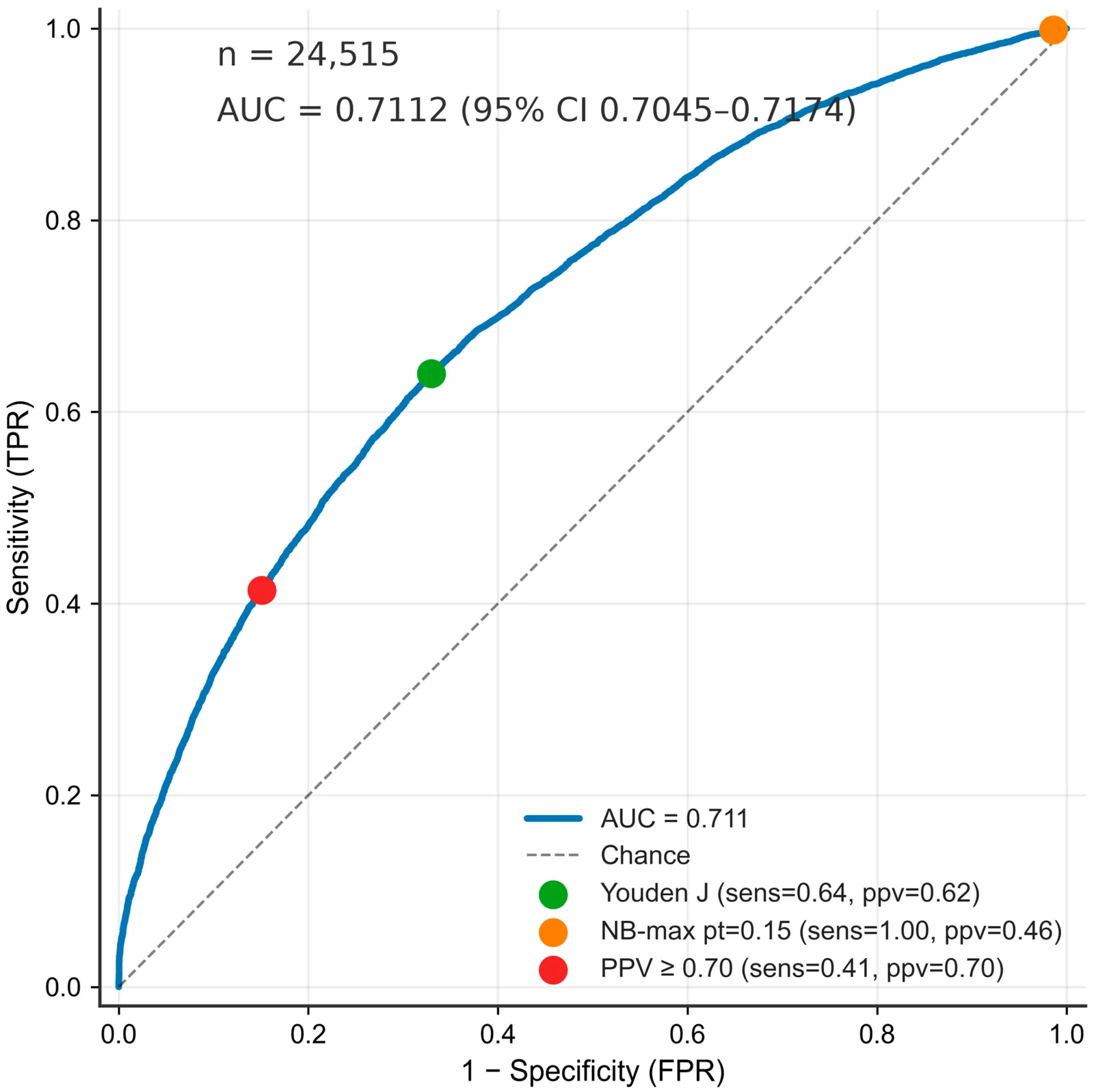
M1 leave-one-athlete-out pooled receiver operating characteristic (ROC) curve on the matched-pair pool (*n* = 24,515), with three pre-specified operating-point markers: Youden-J (balanced-misclassification anchor; sensitivity of 0.640, positive predictive value of 0.623), PPV ≥ 0.70 (deployment anchor; sensitivity of 0.414, positive predictive value of 0.700), and NB-max at $p_{t}$ = 0.15 (decision curve anchor; sensitivity of 0.999, positive predictive value of 0.463). Pooled AUC = 0.7112 (95% bootstrap confidence interval: 0.7045 to 0.7174). The dashed diagonal indicates chance discrimination. Operating-point coordinates are tabulated in Table 6.

Three pre-specified operating points along the M1 ROC curve are reported in Table 6 and Figure 6. Youden-J at *p* = 0.428 yielded sensitivity of 0.640, specificity of 0.670, PPV of 0.623, and NPV of 0.687 (1.36-fold uplift over prevalence). A PPV ≥ 0.70 at *p* = 0.543 yielded PPV of 0.700 with sensitivity of 0.414 and specificity of 0.849 (1.52-fold uplift). The NB-max at the $p_{t}$ = 0.15 operating point is reported alongside the practical-utility check below.

Lift = PPV/pooled prevalence (0.4594). Pooled discrimination metrics: AUC = 0.7112 (95% CI: 0.7045 to 0.7174); AUPRC = 0.6812 (0.6736 to 0.6889); calibration slope = 0.983; calibration intercept = −0.003. The NB-max at the $p_{t}$ = 0.15 operating point is structurally degenerate in a base-rate-balanced setting at this prevalence (see the practical-utility check discussion below) and is reported for transparency rather than as a recommended deployment threshold; the headline practitioner-relevant operating points are Youden-J and PPV ≥ 0.70.

**Figure 7:**
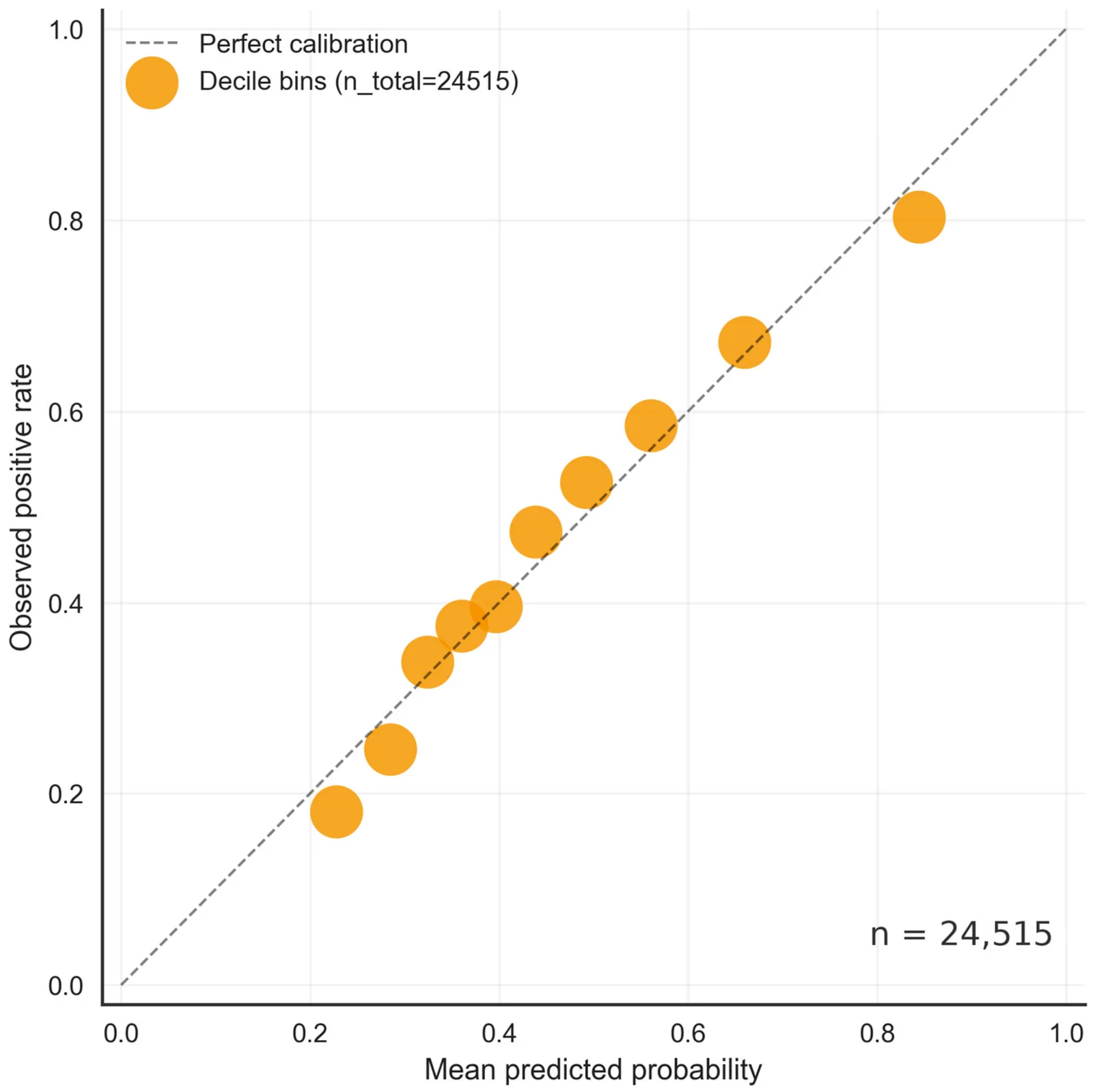
Calibration of M1 leave-one-athlete-out pooled predicted probabilities against decile-bin observed positive rates on the matched-pair pool (*n* = 24,515). The dashed diagonal indicates perfect calibration. Decile-bin observed rates closely track the perfect-calibration diagonal across the predicted-probability range from approximately 0.22 to 0.85. Pooled calibration slope = 0.983; pooled calibration intercept = −0.003. Marker size encodes per-decile sample size ($n_{\mathrm{decile}}\approx 2450$ each).

The pooled calibration slope was 0.983 and pooled calibration intercept was −0.003. Decile-bin observed rates tracked the perfect-calibration diagonal across the predicted-probability range from approximately 0.22 to 0.85, with no systematic over- or under-prediction (Figure 7).

Two reference-noise sensitivity sub-analyses operationalized the First-derivative-as-noisy-reference framing of Section 2.3. Variant 1 (Gaussian noise on FD onset at σ ∈ {5, 10} ms) produced AUCs of 0.7106 at σ = 5 ms and 0.7088 at σ = 10 ms, with a maximum drop of 0.0024 from the unperturbed reference, well below the 0.05 Hopkins [27] trivial-versus-small boundary; the reference-robustness check (Gaussian-noise variant) was not fired. Variant 2 (LOMO-consensus reference) produced AUC = 0.7669, an improvement of 0.0557 versus the First-derivative reference; the reference-robustness check (consensus-reference variant) was not fired. The Variant 2 improvement indicates that the rule’s discrimination is anchored on a method-invariant rocking signal rather than on First-derivative-specific idiosyncrasies, supporting the mechanism-anchored design of the predictor set.

**Figure 8:**
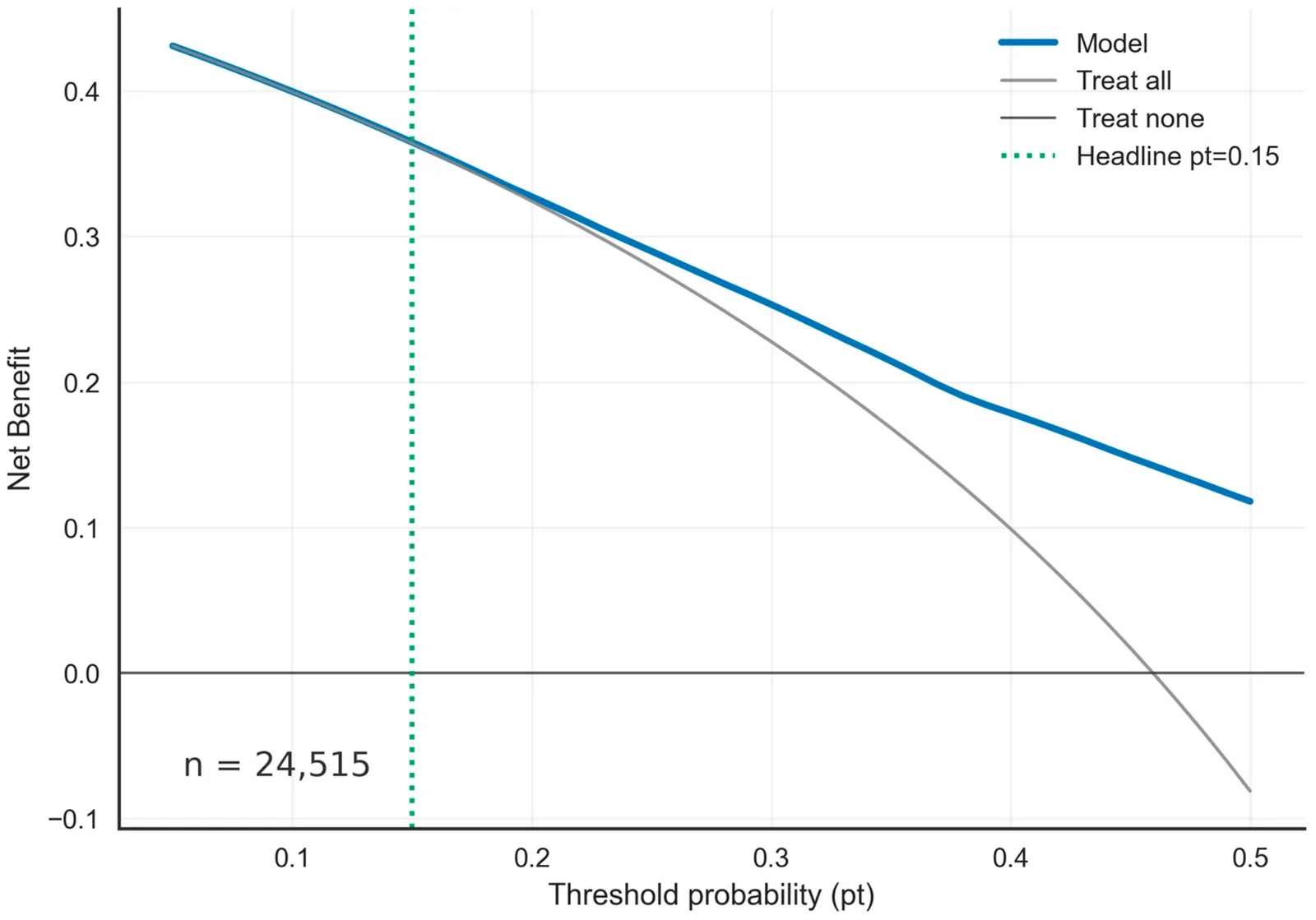
M1 leave-one-athlete-out net-benefit curve on the matched-pair pool (*n* = 24,515), reporting net benefit as a function of threshold probability $p_{t}$ across the operationally relevant range $p_{t}$ ∈ [0.05, 0.50]. The thick line shows the model’s net benefit; the thin reference lines show “treat all” and “treat none”. The vertical dotted line marks the headline $p_{t}$ = 0.15 operating point. At low $p_{t}$ the model line converges by construction toward the treat-all reference because the false-positive penalty weight $p_{t}/$(1 − $p_{t}$) is small at low $p_{t}$; this convergence is the structural basis for the practical-utility check reframing reported above and the recommendation that NB-max at $p_{t}$ = 0.15 is not a practitioner-relevant operating point at this base-rate prevalence.

The practical-utility check tests whether the PPV at NB-max at $p_{t}$ = 0.15 falls below 0.50 with a PPV-to-prevalence ratio below 2.0. The observed PPV at this operating point was 0.4626 and the PPV-to-prevalence ratio was 1.007; the PPV gate was satisfied and the practical-utility check fired. The firing is constructively reframed rather than treated as substantive refutation of practical utility. At the prevailing Label B prevalence of 0.4594, the rule operates in a base-rate-balanced classification setting in which net-benefit-maximization at low threshold probability is structurally degenerate. The false-positive penalty weight $p_{t}/\left(1-p_{t}\right)$ is small at low $p_{t}$, so the net-benefit curve converges by construction toward the treat-all reference [22,23,24]. At $p_{t}$ = 0.15 the selected probability threshold ($\hat{p}$ = 0.184) flags approximately 99% of trials, with sensitivity of 0.999, specificity of 0.014, and PPV approaching the prevalence floor of 0.4594 (Figure 8). The structural reframing converts the practical-utility check firing from an apparent rule failure into a methodological observation about which operating points are appropriate for high-prevalence binary outcomes in sports biomechanics decision rules. The practitioner-relevant operating points for this rule are therefore Youden-J for routine deployment and PPV ≥ 0.70 for high-confidence flagging; NB-max at $p_{t}$ = 0.15 is structurally degenerate at this prevalence and is not recommended (Supplementary Section S3 provides the technical derivation at the prevailing Label B prevalence).

Across the four pre-specified checks, three did not fire and one (the practical-utility check) fired with the constructive structural reframing reported above. The rule’s discrimination falls in the acceptable range of the Hosmer et al. [30] ladder, its calibration is near-perfect across the predicted-probability range, and its reference-noise robustness is supported on both the Gaussian-noise and LOMO-consensus sensitivity sub-analyses [30].

## 4. Discussion

Bilateral weight rocking during pre-jump quiet standing produced false-early-onset firing in approximately four of every nine trials at the operational Δ = 30 ms threshold, with the rocking-amplified subset showing a Range-BW-versus-first-derivative jump height bias approximately 4.7-fold times the dataset-wide bias reported in Adlou et al. [21]. Of the five pre-specified hypotheses, three were supported (H1 incidence; H3 directional jump height bias for Range-BW; H5 decision-rule discrimination) and two were not supported in their original form. H2, which predicted that Range-BW would be the most false-early-prone threshold method, was not supported on either rocking axis, because Range-BW was the least false-early-prone method (Table 2 and Table S3); its rocking-induced bias arose from firing late rather than early, as the mechanism analysis below sets out. The associated analysis nonetheless confirmed the surrogate-axis distinction that motivated the comparison and identified the mechanistically distinctive Range-BW behavior as its bias direction rather than its firing frequency. H4, the sport-level association between Poor-QS proportion and rocking incidence, was not statistically supported (Section 3.3). The multivariable decision rule achieved acceptable discrimination on the Hosmer [30] ladder with near-perfect calibration and was robust to operational-reference-method choice. Three of four pre-specified checks did not fire; the practical-utility check fired and was constructively reframed as a structural property of operating-point selection in base-rate-balanced classification rather than as a substantive refutation of rule utility.

The per-method sign asymmetry in Section 3.4 refines the aggregate jump height bias mechanism described in Adlou et al. [21]. The original statement, that false-early-onset firing extends the integration window and biases jump height upward, holds for Range-BW but inverts for the four other threshold methods, which produce aggregate negative biases (Table 4). The mechanism is method-specific. Range-BW thresholds against the maximum-minus-minimum range of summed vertical force across the quiet-standing window; on rocking trials this range is elevated by the bilateral-force-share oscillation’s coupling to summed vertical force, and Range-BW fires after true unweighting onset, skipping the early below-body-weight portion and biasing jump height upward. The 5 × SD, 4 × SD, Percentage-BW, and Absolute 20 N methods threshold against envelopes crossed during the rocking perturbation itself rather than at true unweighting; they fire earlier than First-derivative on rocking trials, extending the integration window into pre-onset samples predominantly below body weight and producing a downward jump height bias. The within-trial sign-consistency dose–response (Section 3.5) supports this account. Within-trial coherence rises with rocking amplitude because rocking induces a coherent perturbation, while per-method aggregate signs differ because Range-BW’s later-firing mechanism is deterministic on rocking trials and the four other methods’ earlier-firing mechanism is dominated by the stochastic phase of rocking at the moment of threshold crossing. Range-BW is therefore the only threshold method evaluated whose rocking-associated jump height bias is consistent in direction under our mechanistic account. Practitioners interpreting jump height differences across onset detection methods on athletes likely to exhibit rocking should be aware that the four other methods carry population-aggregate negative biases that mask substantial within-trial sign variation.

To place the +0.943 cm rocking-associated jump height difference in a monitoring context, reported test–retest typical error for countermovement jump height in athlete populations is approximately 0.3 to 1.0 cm and the smallest worthwhile change is approximately 1.3 to 1.5 cm [25,26]. The rocking-associated difference therefore exceeds the typical measurement error and is roughly two-thirds of the smallest worthwhile change. A systematic difference of this magnitude, introduced solely by the choice of onset detection method, is large enough to mask or to mimic a genuine worthwhile change when the onset method is not held constant across an athlete’s monitoring timeline. This is the practical hazard the decision rule is intended to flag, and it argues for holding the onset method fixed within a monitoring program.

The pre-specified Spearman rank correlation between per-sport Poor-QS proportion and per-sport any-method false-early-onset rate was not statistically supported (ρ = 0.218, *p* = 0.435; Section 3.3); the positive sign was directionally consistent but non-significant. Two anomalies, Women’s Basketball (low Poor-QS, highest rocking incidence) and Volleyball (high Poor-QS, moderate rocking incidence), indicate that Poor-QS proportion is one of several modifiers of per-sport rocking incidence rather than a dominant predictor. Cohort-specific factors not captured by Poor-QS, including sport-protocol differences in pre-jump cadence, athlete population body mass effects on threshold magnitude sensitivity, and jumping volume effects on within-session fatigue, plausibly contribute to the residual sport-level variation. The 5.19-percentage-point male–female incidence difference cannot be interpreted as a within-sport sex effect, because the 12 sports partition into wholly disjoint men-only and women-only programs; the contrast is labeled by sex between sports. Recent NCAA Division I CMJ comparisons [31,32] have cataloged sport-specific kinetic differences but have not addressed pre-jump rocking as a methodological covariate; the per-sport rocking incidences reported here are the first published values to our knowledge for this population. The non-monotonic cross-sampling-rate pattern (Section 3.3) argues against a simple acquisition-resolution explanation for the false-early phenomenon, which persists across 250, 500, and 1000 Hz and therefore behaves as a physical signature of pre-jump quiet standing rather than an acquisition-rate artifact. Higher sampling rates provide finer onset-time resolution relative to the 30 ms operational threshold, but the rocking signature is present at every rate. The interaction between upstream anti-aliased decimation and the rocking signal is a separate question that is the subject of ongoing work.

The multivariable decision rule’s discrimination and near-perfect calibration support its use as a per-trial decision aid for selecting between Range-BW and First-derivative onset on rocking-amplified trials. Variant 1 (Gaussian noise on the First-derivative reference) produced a maximum AUC drop well below the trivial-versus-small effect-size boundary [27], and Variant 2 (leave-one-method-out consensus reference) improved discrimination relative to the First-derivative reference. The Variant 2 improvement indicates that the rule’s discrimination is anchored on a method-invariant rocking signal rather than on First-derivative-specific idiosyncrasies, supporting the mechanism-anchored design of the predictor set (three rocking-detection signals plus quiet-standing window duration plus quiet-standing tier). Leave-one-sport-out evaluation (Supplementary Table S8) showed discrimination at or above the full-pool benchmark for most sports but more than 0.10 AUC below it in Gymnastics (0.59) and in the combined low-sample golf fold (0.60). The Gymnastics result is mechanistically coherent rather than a failure, because gymnasts exhibit the cleanest quiet standing in the cohort (1.55% Poor-QS), so rocking is rare and the rule has little signal to discriminate. The rule is therefore most useful in populations where pre-jump rocking is prevalent and least informative where it is rare. The rule enters Candidate B as a continuous predictor and does not depend on the tertile cutpoints (6.18 and 18.14 N), which are used only for the descriptive cross-tabulations (Table 2, Figure 3, and Supplementary Table S2). The cutpoint values and the per-sport rocking incidences are specific to this cohort and force-plate platform, and would require recalibration in other datasets, populations, or sensor systems.

The pre-specified practical-utility check fires on the PPV gate at the NB-max at $p_{t}\;$= 0.15 operating point (PPV = 0.463; PPV-to-prevalence ratio = 1.007). The firing is constructively reframed as a structural property of operating-point selection in base-rate-balanced classification rather than as a substantive refutation of practical utility. At Label B prevalence near 0.46, the net-benefit curve at low threshold probability converges by construction toward the treat-all reference because the false-positive penalty weight is small at low $p_{t}$ [22,23,24]. The NB-max at $p_{t}$ = 0.15 operating point in this regime selects a probability threshold ($\hat{p}$ = 0.184) that flags approximately 99% of trials with PPV converging to the prevalence floor. This degeneracy is a property of the decision curve denominator at low $p_{t}$ and high outcome prevalence, not a property of the underlying probabilistic model. Operating-point selection for sports biomechanics decision rules with high-prevalence binary outcomes should therefore favor balanced-misclassification anchors (Youden-J; [29]) for routine deployment and high-confidence flagging anchors (PPV ≥ 0.70) for high-stakes applications. The application of decision curve analysis to force-plate-derived CMJ outcomes is novel in this body of literature; the closest sports medicine precedents [33] and [34] applied decision curve analysis to NBA muscle strain prediction and lower-extremity non-contact injury prediction respectively, neither of which addressed force-plate-derived CMJ kinetics or the high-prevalence net-benefit degeneracy described here.

Within-trial onset disagreement is large in absolute terms (Section 3.5) and is not monotonically driven by rocking magnitude on either axis. The 134 ms median spread exceeds the operational Δ = 30 ms threshold more than four-fold, indicating that practitioner-level inter-method onset disagreement is itself a finding of practical magnitude beyond the rocking phenomenon. Practitioners selecting an onset method for use with bilateral 1D vertical-force-only force plates should be aware that method choice can shift the computed onset by hundreds of milliseconds even outside the rocking-amplified subset. The single-team-to-all-teams Spearman reversal observed in the Volleyball pilot (Supplementary Table S6) further supports the all-teams-headline convention adopted throughout this study.

Two sensor-class design implications follow from these findings. First, the rocking artifact characterized here is observable only because the bilateral plate architecture exposes the force-share asymmetry through Candidate A; sensor designs that report only the summed vertical force lose this diagnostic channel, leaving rocking detectable only through the vertical-axis surrogate Candidate B. Second, sensor pipelines that apply anti-aliased decimation upstream of onset detection add a further interaction with the rocking signal for threshold-based methods. Triaxial force-plate replication would resolve the unobserved anterior–posterior component of rocking and enable a complete sensor-validity account of CMJ onset detection.

### Limitations

Several limitations should be considered. First, VALD ForceDecks FDMax bilateral force plates acquire only the vertical-axis ground reaction force on each plate and report their sum as $F_{z,\mathrm{total}}$. Pure anterior–posterior postural sway with negligible vertical coupling is largely invisible to this sensor class, and the rocking-detection signals of Section 2.2 capture only the medio-lateral and vertical-axis components of rocking. A complementary triaxial laboratory force-plate study would resolve whether the rocking observed here is dominated by medio-lateral, vertical, or anterior–posterior components, and whether anterior–posterior-dominant rocking produces method-specific bias of comparable magnitude. Second, the dataset originates from a single athletics department; the generalizability of per-sport rocking incidence to other Division I institutions, Division II/III collegiate populations, or non-collegiate athlete populations is not established and should be evaluated in follow-up work. The 12-sport diversity within this dataset partially mitigates this limitation but does not eliminate it. Because all records derive from a single bilateral vertical-axis force-plate platform (VALD ForceDecks FDMax), transfer to other force-plate systems with different native sampling and filtering is likewise unestablished. Third, the First-derivative reference is itself susceptible to onset detection noise. The Variant 1 Gaussian-noise sensitivity and Variant 2 LOMO-consensus sensitivity sub-analyses confirm that the decision rule’s discrimination is robust to plausible reference method noise but cannot establish absolute correctness in the absence of manually annotated ground-truth onsets. Fourth, the per-sex contrast in any-method false-early-onset incidence is a between-sport contrast labeled by sex rather than a within-sport effect; isolating a within-sport sex effect would require a dataset with sex-balanced rosters within sports. Fifth, the M3 restricted-cubic-spline specification was disqualified due to numerical instability in the spline basis rather than a substantive functional-form refutation; future replications should evaluate whether a more numerically stable spline implementation produces a meaningfully different operating point than the linear logistic specification reported here. Sixth, many analyses are performed at the trial level while trials are clustered within athletes, so the effective sample size is smaller than the nominal trial count. The headline incidence and jump height results were confirmed under an athlete-clustered bootstrap and the decision rule was cross-validated by athlete, but trial-level confidence intervals reported elsewhere should be read as lower bounds on the true uncertainty.

## 5. Conclusions

Bilateral weight rocking during pre-jump quiet standing produced false-early-onset firing in approximately 46% of countermovement jump trials at the operational Δ = 30 ms threshold on bilateral 1D vertical-force-plate hardware. Among the five threshold-anchored onset detection methods evaluated, only Range-BW produces a positive jump height bias that is consistent in direction on rocking-affected trials; the four other threshold methods exhibit population-aggregate bias in the opposite direction. A multivariable decision rule combining three rocking-detection signals with quiet-standing descriptors achieved acceptable discrimination (AUC = 0.7112) and near-perfect calibration under leave-one-athlete-out cross-validation, with practitioner-relevant operating points at Youden-J for routine override decisions and a PPV ≥ 0.70 for high-confidence flagging. The rule’s discrimination is robust to operational-reference-method choice, and the constructive net-benefit degeneracy reframing of the practical-utility check extends the operating-point-selection literature for sports biomechanics decision rules with high-prevalence binary outcomes.

## Figures and Tables

**Table 1:** Sample characteristics by sport. Trial counts, athlete counts, sex assignment, and quiet-standing tier composition for the *n* = 32,891 incidence pool used in Section 3.1, Section 3.2 and Section 3.3. Sex coding follows team–program–roster designation. Diving and Track athletes are sex-unresolved at the individual level.

Sport	Trials (n)	Athletes	Sex	Good-QS %	Acceptable-QS %	Poor-QS %
Baseball	11,064	111	M	47.7	24.8	27.5
Football	4448	176	M	43.1	26.7	30.2
Gymnastics	3875	32	W	95.3	3.1	1.5
Softball	3543	45	W	88.4	6.7	4.8
Volleyball	2854	32	W	54.3	16.5	29.2
Soccer	1826	50	W	80.4	11.9	7.7
WTennis	1547	10	W	91.5	3.8	4.7
WBasketball	1183	14	W	85.6	9.6	4.7
Track	897	19	U	79.7	14.6	5.7
Diving	753	16	U	74.1	16.7	9.2
MTennis	378	12	M	64.3	9.0	26.7
WSwimming	194	25	W	78.9	11.3	9.8
MSwimming	146	18	M	83.6	11.6	4.8
MGolf	103	8	M	85.4	7.8	6.8
WGolf	80	11	W	90.0	7.5	2.5
**All teams**	**32,891**	**579**		**65.1**	**16.7**	**18.2**

Sex codes: M = men’s program; W = women’s program; U = sex-unresolved. Bold indicates the pooled total across all teams. Athlete counts sum to 579 unique IDs. Total trials = 32,891 after the QS ≥ 1.5 s filter. Cell percentages may not sum to 100 due to rounding.

**Table 2:** Per-method false-early-onset incidence. Pooled false-early-onset rate at the primary Δ = 30 ms threshold and at the secondary Δ = 50 ms sensitivity threshold, with 95% bootstrap confidence intervals. The *n* column reports the per-method denominator after R4/R5 plausibility filtering on the canonical sliding min-SD body weight row. The First-derivative reference contributes 0.00% by construction.

Onset Method	*n*	Δ = 30 ms Rate (95% CI)	Δ = 50 ms Rate (95% CI)
First-derivative (10 Hz LP)	32,891	0.00 (–)	0.00 (–)
Range-BW at 2.5%	32,891	7.67 ([7.39, 7.96])	7.43 ([7.16, 7.71])
Percentage-BW at 2.5%	32,891	28.10 ([27.61, 28.58])	27.66 ([27.18, 28.16])
Absolute 20 N envelope	32,891	28.58 ([28.09, 29.07])	28.15 ([27.66, 28.64])
5 × SD with 30 ms back-step	32,891	29.24 ([28.75, 29.73])	28.85 ([28.36, 29.33])
4 × SD without back-step	32,891	38.07 ([37.54, 38.59])	37.55 ([37.03, 38.06])
**Any-method (pooled)**	**32,891**	**45.89 ([45.4, 46.4])**	**45.32 ([44.8, 45.9])**

The any-method row reports the pooled fraction of trials in which at least one of the five non-first-derivative methods fired more than Δ earlier than the trial’s First-derivative reference. Confidence intervals are 1000-replicate bootstrap percentile intervals on the trial-level denominator. The First-derivative reference contributes 0.00% by definition; this is a definitional consequence of the comparator choice (Section 2.3), not an empirical claim about absolute correctness. Bold indicates the pooled any-method summary row.

**Table 3:** Per-sport rocking incidence and quiet-standing tier composition. Per-sport any-method false-early-onset rate at Δ = 30 ms, per-sport Range-BW false-early-onset rate, and Poor-QS proportion for the *n* = 32,891 incidence pool. The pre-specified Spearman rank correlation between Poor-QS proportion and any-method false-early-onset rate across the 15 sports was ρ = 0.218 (95% CI: −0.34 to +0.72; *p* = 0.435). H4 is not statistically supported; the positive sign is directionally consistent but the correlation is weak and non-significant.

Sport	Trials (n)	Any-Method (%)	Range-BW (%)	Poor-QS (%)
WBasketball	1183	56.21	8.79	4.73
Football	4448	55.51	10.27	30.19
MTennis	378	54.76	7.14	26.72
MGolf	103	50.49	8.74	6.80
Baseball	11,064	46.32	10.12	27.48
WTennis	1547	45.96	5.69	4.65
Gymnastics	3875	44.00	6.37	1.55
Soccer	1826	43.45	4.00	7.67
Volleyball	2854	43.45	2.45	29.15
Track	897	41.92	4.46	5.69
Softball	3543	38.72	6.69	4.83
WSwimming	194	30.41	0.52	9.79
WGolf	80	26.25	1.25	2.50
Diving	753	23.51	5.84	9.16
MSwimming	146	20.55	3.42	4.79

Sports ordered by descending any-method false-early-onset rate. Pearson sensitivity correlations and the corresponding Range-BW column analyses are reported in Supplementary Table S7.

**Table 4:** Per-method paired Δ–jump height summary on the Candidate-A-high subset showing the median, mean, and signed-rank significance of paired Range-BW versus First-derivative jump height differences in the rocking-affected Candidate-A-high subset (*n* = 7262 for all methods; matched-pair pool). Only Range-BW produces an aggregate positive jump height bias; the four other threshold methods produce aggregate negative biases.

Onset Method	*n*	Median ΔJH (cm)	Mean ΔJH (cm)	Wilcoxon p
Range-BW	7262	+0.342	+0.589	<0.001
5 × SD with 30 ms back-step	7262	−0.159	−0.374	<0.001
4 × SD without back-step	7262	−0.098	−0.263	<0.001
Percentage-BW at 2.5%	7262	−0.217	−0.543	<0.001
Absolute 20 N envelope	7262	−0.215	−0.533	<0.001

Paired ΔJH = (method M jump height) − (First-derivative jump height) on the same trial under the canonical sliding min-SD BW row. Median and mean share a common sign within each method. The Wilcoxon signed-rank tests against the null of zero median paired difference were extreme for all five methods (*p* < 10^−90^ for all five; reported as *p* < 0.001 here). The per-method sign asymmetry (Range-BW positive and the other four methods negative) is the empirical seed for the within-trial sign-consistency analysis of Section 3.5.

**Table 5:** Sign-inconsistency proportion per amplitude bin. Proportion of trials with within-trial sign-inconsistent paired Δ–jump height across the five non-first-derivative methods, stratified by the Candidate-A- and Candidate-B-amplitude tertile bin. The proportion decreases monotonically with rocking amplitude on both axes, indicating that within-trial coherence across the multi-method ensemble rises with rocking magnitude.

Axis	Bin	*n* Trials	Sign-Inconsistent (n)	Sign-Inconsistent (%)
Candidate A	low	9225	6179	66.98
Candidate A	medium	8028	4898	61.01
Candidate A	high	7262	3837	52.84
Candidate B	low	9577	6328	66.07
Candidate B	medium	7797	4511	57.86
Candidate B	high	7141	4075	57.06

The dose–response of within-trial sign inconsistency reconciles the per-method aggregate asymmetry of Section 3.4 with the within-trial coherence reported here. Range-BW fires later than First-derivative on rocking trials, skipping the early below-body-weight portion of true unweighting and biasing computed jump height upward. The four other threshold methods fire earlier than First-derivative on rocking trials, extending the impulse–momentum integration window into pre-onset samples that are predominantly below body weight on rocking-affected trials and producing a net subtractive impulse contribution that biases jump height downward. Within-trial sign consistency rises with rocking amplitude because rocking induces a coherent within-trial perturbation pattern. Per-method aggregate signs differ because Range-BW’s later-firing mechanism on rocking trials is more deterministic than the other four methods’ earlier-firing mechanism, the latter dominated by the stochastic phase of rocking at the moment of threshold crossing.

**Table 6:** M1 decision-rule operating-point coordinates on the leave-one-athlete-out pooled ROC curve. Three pre-specified operating points are reported with predicted-probability threshold $\hat{p}$ and per-point sensitivity, specificity, positive predictive value (PPV), negative predictive value (NPV), F1, and number of positively predicted trials ($n_{\mathrm{pos}\;\mathrm{pred}}$) on the matched-pair pool (*n* = 24,515). Pooled Label B prevalence is 0.4594.

Operating Point	$\hat{p}$	Sens	Spec	PPV	NPV	F1	$n_{\mathrm{pos}\;\mathrm{pred}}$	Lift
Youden-J	0.428	0.640	0.670	0.623	0.687	0.631	11,577	1.36
PPV ≥ 0.70	0.543	0.414	0.849	0.700	0.630	0.520	6660	1.52
NB-max at *p_t_* = 0.15	0.184	0.999	0.014	0.463	0.930	0.632	24,315	1.01

Lift = PPV/pooled prevalence (0.4594). Pooled discrimination metrics: AUC = 0.7112 (95% CI: 0.7045 to 0.7174); AUPRC = 0.6812 (0.6736 to 0.6889); calibration slope = 0.983; calibration intercept = −0.003. The NB-max at the *p_t_* = 0.15 operating point is structurally degenerate in a base-rate-balanced setting at this prevalence (see the practical-utility check discussion below) and is reported for transparency rather than as a recommended deployment threshold; the headline practitioner-relevant operating points are Youden-J and PPV ≥ 0.70.

## Data Availability

Data will not be publicly available according to the department’s policies, but will be available upon reasonable request and with case-by-case approval from the department.

## Supplementary Materials

The following supporting information can be downloaded at https://www.mdpi.com/article/10.3390/s26165043/s1: Section S1: Inherited processing pipeline; Section S2: Pre-specified falsification checks; Section S3: Net-benefit convergence at high outcome prevalence; Section S4: Leave-one-sport-out portability of the decision rule; Table S1: Pre-specified falsification checks with cut-points, anchoring references, and rationale. A check is said to be met when its cut-point condition is satisfied; a met check indicates pre-specified refutation of the associated hypothesis or quality criterion; Table S2: Per-method false-early-onset incidence at Δ = 30 ms binned by Candidate-B tertile across the n = 32,891 incidence pool. Tertile cut-points were 6.18 N and 18.14 N (bin n = 10,960/10,972/10,959). Cells are percentages with 95% bootstrap confidence intervals; Table S3: Per-method paired phase-duration and takeoff-velocity differences on the Candidate-A-high subset (n = 7262 matched-pair trials). Each cell reports median (mean) of (method M value) − (First-derivative value) on the same trial under the canonical Sliding min-SD BW row; Table S4: Model and reference net benefit across threshold probabilities pt at positive-label prevalence p = 0.4594. Treat-all NB = p − (1 − p) × $p_{t}$ / (1 − $p_{t}$); treat-none NB = 0. The model-NB column reports the decision rule’s net benefit at each pt (net-benefit-optimal threshold convention, reproducing the Figure 8 curve and the pt = 0.15 worked example; the strict threshold-equals-pt convention differs by ≤ 0.004 and is reported in the text); Table S5: Body weight estimator sensitivity of the Range-BW versus First-derivative jump-height difference on the Candidate-A-high subset. Median and mean paired difference and Wilcoxon signed-rank significance against zero under each estimator. The Sliding min-SD row is the canonical estimator reported in the main manuscript; Table S6: Spearman rank correlations between within-trial onset spread (ms) and rocking-surrogate magnitude on both axes, for the full matched-pair pool (n = 24,515) and the Candidate-B-high subset (n = 7141); Table S7: Pearson sensitivity correlations across the 15 sports for the H4 relationship. Pearson r and approximate two-sided p-value (from t = r × √(n − 2) / √(1 − r²) at n = 15) for the any-method row and the Range-BW row against per-sport Poor-QS proportion; Table S8: Leave-one-sport-out portability of the decision rule. Per-held-out-sport pooled AUC with 95% bootstrap confidence interval and difference from the full-pool AUC (0.7112). Sample sizes are matched-pair trials in the held-out fold (a subset of n = 24,515).
