# Supplementary material for: A Multivariable Decision Rule for Weight-Rocking-Driven Onset Detection Method Bias on Bilateral Force Plates Across 32,952 Countermovement Jump Trials
Source: Sensors (Basel). 2026 Aug 8;26(16):5043. doi: 10.3390/s26165043 (PMC13517217; doi:10.3390/s26165043)
Supplement: Supplementary file 1 [file sensors-26-05043-s001.zip › sensors-4378010-supplementary.pdf]

Supplementary Material

A Multivariable Decision Rule for Weight-Rocking-Driven Onset Detection Method Bias On Bilateral Force Plates Across 32,952 Countermovement Jump Trials

Bahman Adlou, Michael D. Goodlett, Christopher Wilburn, Wendi Weimar

Contents

S1. Inherited processing pipeline (self-contained summary)
S2. Pre-specified falsification checks (Table S1)
Table S2. Candidate-B-binned per-method false-early-onset incidence
Table S3. Phase-duration and takeoff-velocity differences on the Candidate-A-high subset
S3. Net-benefit convergence at high outcome prevalence (Table S4)
Table S5. Body weight estimator sensitivity of the jump-height difference
Table S6. Within-trial onset spread versus rocking-surrogate magnitude
Table S7. Sport-level correlation sensitivity check (Pearson)
S4. Leave-one-sport-out portability of the decision rule (Table S8)

S1. Inherited processing pipeline

To make the present study self-contained, this section summarizes the upstream trial-segmentation, body weight (BW) estimation, onset-detection, quiet-standing (QS) quality classification, and plausibility-filtering procedures inherited from the companion methods paper (Adlou et al., in review). The companion paper provides the full derivation and validation; the summary here is sufficient to reproduce the analyses reported in the main manuscript without access to that paper.

Trial segmentation and the quiet-standing window. Each trial was segmented from the continuous summed vertical ground reaction force (vGRF) record to identify a movement-onset landmark. The pre-movement quiet-standing window for a trial is the half-open interval from the first available sample to this segmentation onset; the three rocking-surrogate signals (Methods 2.2) and the QS quality tier are computed within this window. The segmentation onset is independent of the six onset-detection methods compared in the main analyses.

Body weight estimators. Five BW estimators were computed per trial from the summed vGRF during quiet standing. Sliding min-SD (canonical) takes the mean force over the 1 s window with the lowest within-window standard deviation. Trimmed-mean (10%) and Median take the corresponding robust central tendency over the quiet-standing window. Fixed 1-s and Fixed 2-s take the mean force over a fixed window of the stated duration immediately preceding the segmentation onset. The Sliding min-SD estimator is the canonical row throughout; Table S5 reports sensitivity of the headline jump-height result to estimator choice.

Onset-detection methods. Six onset-detection methods were applied. First-derivative applies a 10 Hz low-pass filter and detects the first sustained departure in the rate of change of force. 5 × SD places the threshold five within-window standard deviations from BW and applies a 30 ms back-step. 4 × SD places the threshold four standard deviations from BW with no back-step. Range-BW sets a bidirectional band at 2.5% of the maximum-minus-minimum force range of the quiet-standing window. Percentage-BW sets the threshold at 2.5% of body weight. Absolute sets the threshold at a fixed 20 N envelope. First-derivative served as the operational onset reference (Methods 2.3).

Quiet-standing quality tier. Each trial was assigned a QS quality tier from the standard deviation of summed vGRF over the 1 s sliding-minimum-SD window: Good (≤ 5 N), Acceptable (5–10 N), Poor (> 10 N). The Section 2 pool distribution was Good 65.12%, Acceptable 16.72%, Poor 18.15%.

Plausibility filtering. A two-stage filter excluded a trial-method row when the engine failed to extract a required landmark (criterion R4) or when a derived quantity fell outside literature-anchored bounds (criterion R5): jump height 0.05–0.80 m, takeoff velocity 0.99–4.0 m s^{-1}, countermovement depth −0.60 to −0.05 m, unweighting duration 50–1500 ms, braking duration 30–600 ms, propulsion duration 50–600 ms. The full processing pipeline was applied to 32,952 trials; two further filters specific to the present study (a 1.5 s minimum quiet-standing duration, and a conservative paired-drop rule for matched-pair analyses) yielded the n = 32,891 and n = 24,515 working pools described in Methods 2.1.

S2. Pre-specified falsification checks

Each hypothesis-driven claim in this study was paired with a falsification check whose cut-point was fixed before analysis and anchored on a published effect-size or discrimination threshold rather than on within-sample tuning. Table S1 lists the seven checks, their cut-points, the anchoring literature, and the rationale. A check is met when its cut-point condition is satisfied; the discrimination, practical-utility, and reference-robustness checks (rows 4–7) bear on the decision rule, of which three were not met and one (practical utility) was met and is interpreted in Section 3.6 and the Discussion of the main manuscript.

Table S1. Pre-specified falsification checks with cut-points, anchoring references, and rationale. A check is said to be met when its cut-point condition is satisfied; a met check indicates pre-specified refutation of the associated hypothesis or quality criterion.

Check	Hypothesis	Cut-point condition	Anchoring reference	Verdict
Incidence floor	H1	Pooled any-method false-early rate < 0.5% at Δ = 30 ms	Substantive practical-relevance floor (1 in 200 trials)	Not met
Magnitude	H3	Median |ΔJH| (Range-BW) < 0.50 cm in Candidate-A-high subset	Conservative sub-SWC floor for CMJ jump height (Pueo 2020; Comyns 2023; Hopkins 2009)	Not met
Direction	H3	Median signed ΔJH (Range-BW) < 0 in Candidate-B-high subset	H3 directional prediction (Adlou et al., in review)	Not met
Discrimination	H5	Pooled AUC < 0.65 with bootstrap upper CI < 0.70	Hosmer et al. (2013) no-discrimination-versus-acceptable ladder	Not met
Practical utility	H5	PPV at NB-max (pt = 0.15) < 0.50 with PPV-to-prevalence ratio < 2.0	Vickers & Elkin (2006); Steyerberg (2019)	Met; interpreted
Reference robustness (Gaussian)	H5	AUC drop > 0.05 under Gaussian noise (σ ∈ {5, 10} ms) on First-derivative	Hopkins (2009) trivial-versus-small boundary on the AUC scale	Not met
Reference robustness (consensus)	H5	AUC drop > 0.05 under leave-one-method-out consensus reference	Hopkins (2009) trivial-versus-small boundary on the AUC scale	Not met

The anchors operate at two scales. Effect-size anchors (the magnitude and reference-robustness checks) translate a smallest-worthwhile-change or trivial-versus-small boundary into a substantive refutation threshold. Discrimination and practical-utility anchors translate published practitioner-relevant performance floors into deployment-relevant refutation thresholds. The direction check is the only one whose anchor is internal to the present study; its cut-point is the directional prediction of H3 itself.

Table S2. Candidate-B-binned per-method false-early-onset incidence

Table 2 of the main manuscript reports per-method false-early-onset incidence binned by Candidate A (medio-lateral force-share oscillation). Table S2 reports the same incidence binned by Candidate B (the vertical-axis sliding-SD signal), the surrogate most directly coupled to summed vGRF and therefore the mechanism-relevant axis for methods that threshold against summed force.

Table S2. Per-method false-early-onset incidence at Δ = 30 ms binned by Candidate-B tertile across the n = 32,891 incidence pool. Tertile cut-points were 6.18 N and 18.14 N (bin n = 10,960 / 10,972 / 10,959). Cells are percentages with 95% bootstrap confidence intervals.

Onset method	Low (%, 95% CI)	Medium (%, 95% CI)	High (%, 95% CI)
First-derivative	0.00 (–)	0.00 (–)	0.00 (–)
Range-BW	6.65 (6.20–7.12)	8.23 (7.75–8.77)	8.13 (7.62–8.61)
Percentage-BW	10.85 (10.30–11.45)	33.02 (32.15–33.93)	40.41 (39.53–41.41)
Absolute 20 N	9.71 (9.18–10.26)	34.70 (33.82–35.59)	41.32 (40.45–42.21)
5 × SD	28.51 (27.71–29.36)	36.69 (35.78–37.57)	22.49 (21.74–23.29)
4 × SD	41.61 (40.59–42.51)	45.69 (44.74–46.58)	26.89 (26.05–27.67)
Any method	41.74 (40.87–42.67)	51.42 (50.53–52.37)	44.51 (43.61–45.47)

The two binning axes are complementary. Percentage-BW and Absolute 20 N rise steeply and monotonically with Candidate B, from roughly 10% in the low tertile to roughly 40% in the high tertile, because both threshold against a fixed or proportional summed-force band that vertical fluctuation pushes the signal across. Range-BW rises modestly but significantly from the low to the high tertile (6.65% to 8.13%; non-overlapping confidence intervals) yet remains the least false-early-prone method on every Candidate-B bin, because its bidirectional band widens as vertical fluctuation increases and therefore tends to relocate its detection later rather than earlier. The standard-deviation methods are highest in the low and medium tertiles and decline in the high tertile, consistent with high within-window fluctuation inflating their standard-deviation-scaled threshold. The mechanism-relevant finding for Range-BW is therefore its bias direction (Table 4 and Section 3.4 of the main manuscript), not its false-early frequency.

Table S3. Phase-duration and takeoff-velocity differences

Table 4 of the main manuscript reports paired jump-height differences between each non-First-derivative method and the First-derivative reference on the Candidate-A-high subset. Table S3 reports the corresponding paired differences for the three phase durations and for takeoff velocity over the same subset.

Table S3. Per-method paired phase-duration and takeoff-velocity differences on the Candidate-A-high subset (n = 7,262 matched-pair trials). Each cell reports median (mean) of (method M value) − (First-derivative value) on the same trial under the canonical Sliding min-SD BW row.

Onset method	ΔUnweighting (ms)	ΔBraking (ms)	ΔPropulsion (ms)	ΔTakeoff velocity ($m\ s^{-1}$)
Range-BW	−96.0 (−116.7)	−1.0 (−1.9)	+1.0 (+1.9)	+0.012 (+0.021)
5 × SD	−40.0 (−15.0)	0.0 (+0.9)	0.0 (−0.9)	−0.006 (−0.013)
4 × SD	−64.0 (−17.6)	0.0 (+0.6)	0.0 (−0.6)	−0.003 (−0.009)
Percentage-BW	−56.0 (+8.2)	0.0 (+1.5)	0.0 (−1.5)	−0.008 (−0.019)
Absolute 20 N	−55.0 (+13.2)	0.0 (+1.4)	0.0 (−1.4)	−0.008 (−0.019)

Braking and propulsion durations are essentially onset-method-invariant (|median| ≤ 1 ms, |mean| ≤ 2 ms): these phases are bounded by force landmarks that do not depend on the onset method, so the small braking and propulsion differences mirror one another around a slightly relocated braking-to-propulsion boundary and are of negligible magnitude. The onset-method effect on phase structure is therefore concentrated almost entirely in the unweighting phase, which begins at the onset. The takeoff-velocity differences share the sign of the corresponding jump-height differences (Table 4), consistent with the impulse-momentum identity that constrains takeoff velocity and jump height to co-vary.

S3. Net-benefit convergence at high outcome prevalence

The practical-utility check was met at the pre-specified net-benefit-maximizing operating point for a threshold probability of pt = 0.15, with observed PPV = 0.4626 and a PPV-to-prevalence ratio of 1.007 (Section 3.6 of the main manuscript). This was interpreted as a structural property of net-benefit maximization at low threshold probability in a base-rate-balanced classification setting rather than as substantive refutation of the rule's practical utility. This section gives the technical derivation at the prevailing positive-label prevalence of p = 0.4594.

Definitions. Net benefit (NB) at threshold probability pt for a model M, binary outcome of prevalence p, on N trials, is NB(M, pt) = (TP / N) − (FP / N) × w(pt), where TP and FP are true- and false-positive counts at pt and w(pt) = pt / (1 − pt) is the false-positive penalty weight. The treat-all reference is NB(treat-all, pt) = p − (1 − p) × w(pt); the treat-none reference is 0 by construction.

Convergence at low pt. As pt → 0, w(pt) → 0 and both NB(M, pt) and NB(treat-all, pt) converge to their first terms (TP / N and p). For a high-sensitivity model at low pt that flags nearly all trials, sensitivity approaches 1 and TP / N approaches p, so the model and the treat-all reference converge to the same NB and their difference becomes vanishingly small relative to the variance of the estimate. At p = 0.4594 this convergence is already observable at pt = 0.15, where the selected probability threshold (0.184) flags approximately 99% of trials (sensitivity 0.999, specificity 0.014; Table 6 of the main manuscript). The numerical worked example gives NB(model, 0.15) ≈ 0.4589 − 0.5330 × (0.15/0.85) ≈ 0.365 and NB(treat-all, 0.15) ≈ 0.4594 − 0.5406 × (0.15/0.85) ≈ 0.364, a difference of about 0.001 net-benefit units, which is not distinguishable at conventional bootstrap-CI widths.

Why this is a property of the decision-curve denominator, not the model. The convergence is a property of the NB calculation at high outcome prevalence and low pt, not of the underlying probabilistic model, whose calibration slope (0.983) and discrimination (AUC 0.7112) are stable across the predicted-probability range. At higher pt, where w(pt) is large enough to separate the model from treat-all, the model's discrimination becomes visible in NB. Table S4 reports the model and reference NB across the practically relevant range; the convergence at low pt and the divergence at high pt are visible numerically.

Table S4. Model and reference net benefit across threshold probabilities pt at positive-label prevalence p = 0.4594. Treat-all NB = p − (1 − p) × p_t / (1 − p_t); treat-none NB = 0. The model-NB column reports the decision rule's net benefit at each pt (net-benefit-optimal threshold convention, reproducing the Figure 8 curve and the pt = 0.15 worked example; the strict threshold-equals-pt convention differs by ≤ 0.004 and is reported in the text).

pt	w(pt) = pt / (1 − pt)	Treat-all NB	Treat-none NB	Model NB
0.05	0.0526	0.431	0	0.431
0.10	0.1111	0.399	0	0.400
0.15	0.1765	0.364	0	0.365
0.20	0.2500	0.324	0	0.327
0.30	0.4286	0.228	0	0.253

pt	w(pt) = pt / (1 − pt)	Treat-all NB	Treat-none NB	Model NB
0.40	0.6667	0.099	0	0.179
0.50	1.0000	−0.081	0	0.118

Treat-all NB becomes negative beyond pt ≈ p = 0.4594, where the false-positive penalty exceeds the prevalence-determined true-positive gain. The model retains positive net benefit across the full range, but at low pt its advantage over treat-all is within estimation noise (the convergence above), while at higher pt its discrimination produces a clear separation. Practitioner-relevant operating points at high outcome prevalence should therefore be selected on balanced-misclassification or PPV anchoring rather than net-benefit maximization at low pt (Section 3.6 and Discussion of the main manuscript).

Table S5. Body weight estimator sensitivity

The headline jump-height result (Table 4 of the main manuscript) was computed under the canonical Sliding min-SD BW row. Table S5 recomputes the paired Range-BW versus First-derivative jump-height difference on the Candidate-A-high subset under each of the five BW estimators. The sample size differs across estimators because of estimator-specific availability (for example, the Fixed 2-s estimator requires 2 s of quiet standing).

Table S5. Body weight estimator sensitivity of the Range-BW versus First-derivative jump-height difference on the Candidate-A-high subset. Median and mean paired difference and Wilcoxon signed-rank significance against zero under each estimator. The Sliding min-SD row is the canonical estimator reported in the main manuscript.

BW estimator	n	Median ΔJH (cm)	Mean ΔJH (cm)	Wilcoxon p
Sliding min-SD (canonical)	7,262	+0.342	+0.589	< 0.001
Trimmed-mean (10%)	7,226	+0.341	+0.581	< 0.001
Median	7,155	+0.345	+0.583	< 0.001
Fixed 1-s	4,560	+0.313	+0.344	< 0.001
Fixed 2-s	5,858	+0.350	+0.449	< 0.001

All five estimators yield a positive Range-BW versus First-derivative jump-height difference (median +0.31 to +0.35 cm; Wilcoxon $p < 0.001$ throughout), so the positive bias direction is not an artifact of the canonical BW choice. Wilcoxon signed-rank p-values were below 10^{-140} for every estimator and are reported as < 0.001.

Table S6. Within-trial onset spread versus rocking-surrogate magnitude

Within-trial onset spread is the maximum minus minimum onset time across the six onset-detection methods within each trial (Section 3.5 of the main manuscript). The spread is large in absolute terms (median 134 ms; 95th percentile 894 ms across the n = 24,515 matched-pair pool) but is not monotonically driven by rocking-surrogate magnitude on either axis.

Table S6. Spearman rank correlations between within-trial onset spread (ms) and rocking-surrogate magnitude on both axes, for the full matched-pair pool (n = 24,515) and the Candidate-B-high subset (n = 7,141).

Correlation	ρ	p	n
Candidate A vs spread (full pool)	−0.092	< 0.001	24,515
Candidate A vs spread (Candidate-B-high)	−0.196	< 0.001	7,141
Candidate B vs spread (full pool)	−0.002	0.742	24,515
Candidate B vs spread (Candidate-B-high)	+0.081	< 0.001	7,141

All four correlations satisfy $|\rho| < 0.20$ with mixed directions. The conventional null was rejected by extreme-tail p-values on the large-sample correlations, but the practical magnitudes are below the trivial-versus-small boundary. Within-trial onset spread is therefore large in absolute terms but not monotonically driven by rocking magnitude on either axis. A single-team pilot of this analysis on the Volleyball team (n = 2,854) returned ρ = +0.325 between

spread and Candidate-B amplitude on within-team data, an estimate that did not replicate at the all-teams scale; the reversal supports the all-teams-headline convention adopted throughout this study.

Table S7. Sport-level correlation sensitivity check (Pearson)

The pre-specified H4 test (Section 3.3 of the main manuscript) was a Spearman rank correlation between per-sport Poor-QS proportion and per-sport any-method false-early-onset rate across the 15 sports ($\rho = 0.218$; 95% CI −0.34 to +0.72; $p = 0.435$). Table S7 reports the Pearson sensitivity check for the same relationships.

Table S7. Pearson sensitivity correlations across the 15 sports for the H4 relationship. Pearson r and approximate two-sided p-value (from $t = r \times \sqrt{(n - 2)} / \sqrt{(1 - r^2)}$ at $n = 15$) for the any-method row and the Range-BW row against per-sport Poor-QS proportion.

Incidence row	Pearson r	Approx. p (two-sided)	n sports
Any-method vs Poor-QS proportion	+0.447	≈ 0.094	15
Range-BW row vs Poor-QS proportion	+0.330	≈ 0.229	15

Both Pearson r values are positive, directionally consistent with the H4 prediction, but neither rejects the conventional null at the 15-sport sample size, the same qualitative outcome as the pre-specified Spearman test. H4 is therefore not statistically supported on either correlation measure; the positive sign is consistent with Poor-QS being a minor modifier rather than a dominant predictor of per-sport rocking incidence.

S4. Leave-one-sport-out portability of the decision rule

Portability across sports was assessed under a leave-one-sport-out (LOSO) scheme: the decision rule was refit on the remaining sports and evaluated on the held-out sport. The procedure yields 14 folds covering all 15 sports, because Men's Golf and Women's Golf each contribute fewer than 100 matched-pair trials and are combined into a single low-sample fold (single-sport estimates at $n < 100$ would be unstable). Held-out AUCs reproduce the locked per-sport values to within 5×10^{-5}; the 95% confidence intervals reported here are a stratified bootstrap on the held-out predictions. The full pooled leave-one-athlete-out AUC of 0.7112 (Section 3.6) is the benchmark.

Table S8. Leave-one-sport-out portability of the decision rule. Per-held-out-sport pooled AUC with 95% bootstrap confidence interval and difference from the full-pool AUC (0.7112). Sample sizes are matched-pair trials in the held-out fold (a subset of n = 24,515).

Held-out fold	Trials (n)	AUC	95% CI	Δ from full pool
Baseball	7,576	0.728	0.717 to 0.739	+0.016
Softball	3,095	0.715	0.697 to 0.732	+0.004
Gymnastics	2,894	0.588	0.566 to 0.609	−0.123
Football	2,882	0.731	0.713 to 0.749	+0.020
Volleyball	2,081	0.759	0.738 to 0.779	+0.047
Soccer	1,485	0.734	0.708 to 0.761	+0.023
WTennis	1,406	0.767	0.741 to 0.791	+0.056
WBasketball	974	0.701	0.670 to 0.737	−0.010
Track	768	0.720	0.682 to 0.758	+0.009
Diving	607	0.698	0.653 to 0.742	−0.013
MTennis	282	0.774	0.714 to 0.826	+0.063
WSwimming	172	0.680	0.586 to 0.758	−0.032
MGolf + WGolf (combined)	158	0.596	0.503 to 0.684	−0.115
MSwimming	135	0.667	0.564 to 0.758	−0.044

Discrimination is preserved at or above the full-pool benchmark for most sports and is highest in sports with substantial rocking incidence. Two folds fall more than 0.10 AUC below the benchmark: Gymnastics (0.588) and the combined golf fold (0.596). The Gymnastics result is mechanistically coherent rather than a failure: gymnasts exhibit the cleanest quiet standing in the cohort (1.55% Poor-QS; Table 3 of the main manuscript), so rocking is rare and the rule has little signal to discriminate. The rule is deployed for per-trial onset-method selection within sport, not for cross-sport prediction, so LOSO is a portability stress test rather than the operational scheme; the pre-specified primary scheme is 20 grouped folds over athlete identifier.
